# Comparative genomics and functional analysis of a highly adhesive dairy *Lactobacillus paracasei* subsp. *paracasei* IBB3423 strain

**DOI:** 10.1007/s00253-019-10010-1

**Published:** 2019-07-29

**Authors:** Anna Koryszewska-Bagińska, Jan Gawor, Adriana Nowak, Marcin Grynberg, Tamara Aleksandrzak-Piekarczyk

**Affiliations:** 1 Institute of Biochemistry and Biophysics, Polish Academy of Sciences (IBB PAS), Pawińskiego 5a, 02-106 Warsaw, Poland; 2 Present Address: Department of Medical Biology, Medical University of Warsaw, Litewska 14/16, 00-575 Warsaw, Poland; 3 Institute of Fermentation Technology and Microbiology, Lodz University of Technology, Wolczanska 171/173, 90-924 Lodz, Poland

**Keywords:** Lactic acid bacteria, *Lactobacillus paracasei*, Adhesion, *spaCBA* pilus genes cluster

## Abstract

Various *Lactobacillus paracasei* strains are found in diverse environments, including dairy and plant materials and the intestinal tract of humans and animals, and are also used in the food industry or as probiotics. In this study, we have isolated a new strain *L. paracasei* subsp. *paracasei* IBB3423 from samples of raw cow milk collected in a citizen science project. IBB3423 showed some desired probiotic features such as high adhesion capacity and ability to metabolize inulin. Its complete genome sequence comprising the chromosome of 3,183,386 bp and two plasmids of 5986 bp and 51,211 bp was determined. In silico analysis revealed numerous genes encoding proteins involved in carbohydrate metabolism and of extracellular localization likely supporting interaction with host tissues. In vitro tests confirmed the high adhesion capacity of IBB3423 and showed that it even exceeds that of the highly adhesive *L. rhamnosus* GG. Curing of the larger plasmid indicated that the adhesive properties depend on the plasmid and thus could be determined by its pilus-encoding *spaCBA* genes.

**Electronic supplementary material:**

The online version of this article (10.1007/s00253-019-10010-1) contains supplementary material, which is available to authorized users.

## Introduction

*Lactobacillus* is the largest genus of the lactic acid bacteria (LAB) group, which currently comprises 236 species listed in the List of Prokaryotic Names (October 2018; www.bacterio.net). Members of this genus represent an extremely diverse group of species with various physiological, biochemical, and genetic characteristics and are able to colonize diverse ecological niches, including the gastrointestinal tract (GIT) of humans and animals, plants, and dairy food environments (Makarova et al. 2006; Kandler 1983). Lactobacilli are rod-shaped, Gram-positive, non-spore-forming, lactic acid-producing, generally nonmotile bacteria that can survive mainly in anaerobic environments (Claesson et al. 2007; Kandler 1983). Due to their advantageous properties and a long history of safe use, lactobacilli are used as starter cultures in both traditional fermentation and various industrial bioprocesses (Stiles and Holzapfel 1997). Furthermore, having the generally recognized as safe (GRAS) status, many species are considered probiotics, i.e., live microorganisms intended to confer health benefits after consumption (Makarova et al. 2006; Fuller 1991; Ouwehand et al. 2002; Lebeer et al. 2008). The probiotic properties of microorganisms are determined by factors that include their adaptation to the GIT and those directly mediating the health effects to the host. The adaptation factors include the ability to survive in the harsh conditions of the GIT (low pH and high concentration of bile salts) and adherence to mucosal surfaces (mucus, extracellular matrix proteins (ECM), or epithelial cells) (Holzapfel et al. 2001; Marco et al. 2006; Lebeer et al. 2008). One of the most desirable traits for probiotic bacteria is good adherence capacity, as it is responsible for successful colonization of the mucosal surfaces (Ouwehand et al. 2000; Styriak et al. 2003). By adhering to the host gut, the probiotic microbiota provides beneficial effects through various mechanisms ensuring the maintenance of gut barrier function, enhancement of a balanced microbial flora, immunomodulation of gut mucosal defenses, and competitive exclusion of pathogens (Jankowska et al. 2008; Lim 2012). The process of adhesion is initially based on non-specific physical interactions between two surfaces (such as van der Waals forces, repulsive electrostatic double-layer forces, hydrophobic interactions, short-range forces, and ion bridging). Generally, hydrophobic cells adhere strongly to hydrophobic surfaces, and thus the phenomenon of cell surface hydrophobicity (CSH) is responsible for cell aggregation, and, as human body surfaces are mostly of a hydrophobic character, it promotes binding of hydrophobic bacteria to the intestine epithelial tissue (Krasowska and Sigler 2014). After the initial attachment to the epithelium surface, a more specific interplay between bacterial adhesion proteins and mucosal surfaces takes place, including binding to specific receptors (Pérez et al. 1998; Turroni et al. 2013). A large number of diverse factors mediating adhesion to different components of the mucosa have been identified largely owing to genome sequencing of various lactobacilli paired with integrated genomic techniques (Kant et al. 2011; Smokvina et al. 2013). However, in contrast to the often well-characterized adhesive determinants of pathogens and their specific host receptors, functional understanding of the adherence of commensal bacteria is only fragmentary. These bacterial adhesive factors include exopolysaccharides (EPSs), teichoic acids (TAs), surface layer (S-layer) proteins, and some other membrane- or cell wall-associated proteins protruding from the cell (Lebeer et al. 2008). Molecules involved in adhesion can be attached to the cell wall by LPXTG (Leu-Pro-X-Thr-Gly)-type anchor or other peptidoglycan-binding domains such as LysM, WxL, SH3, or PG (Kleerebezem et al. 2010; Visweswaran et al. 2014). Most adhesins of lactobacilli belong to a class of sortase-dependent proteins (Velez et al. 2007), which contain an N-terminal signal sequence for transport through the membrane and a C-terminal motif LPXTG for cell wall anchoring performed by the enzyme sortase (Navarre and Schneewind 1999). In Gram-positive pathogens, the SpaCBA pili employing an LPXTG-like motif are the major driver of adhesion (Mandlik et al. 2008). Similar structures have been also identified in many lactobacilli, for example, *L. rhamnosus* GG and *L. paracasei* LOCK 0919 (Kankainen et al. 2009; Aleksandrzak-Piekarczyk et al. 2016).

To interact with host receptors, bacterial adhesion proteins contain specific domains, among them are the following: mucin binding (MucBP), collagen binding (CnaB, collagenBindB, collagen_bind), fibronectin binding (FbpA), leucine-rich repeats (LRR), Ig-like (Big_3, BID_2), legume lectin-like (lectin L-type), and ZnuA (Barr et al. 2013; Dintilhac et al. 1997; Gabbianelli et al. 2011). Of those, the following have been studied in some detail: fibronectin-binding proteins in *Lactobacillus casei* BL23 (Muñoz-Provencio et al. 2010), collagen-binding surface proteins in *L. reuteri* NCIB11951 and *L. acidophilus* NCFM (Aleljung et al. 1994; Buck et al. 2005), mucus-adhesion-promoting protein (MapA) in *L. reuteri* (Miyoshi et al. 2006), and lectin-like mannose-specific adhesin in *L. plantarum* WCFS1 (Pretzer et al. 2005). A key role in adhesion to intestinal cells has also been established for so-called S-layer proteins in a number of lactobacilli such as *L. brevis* (Hynonen et al. 2002), *L. crispatus* (Toba et al. 1995; Antikainen et al. 2002), *L. helveticus* (Johnson-Henry et al. 2007), and *L. acidophilus* (Buck et al. 2005). Some probiotic strains devoid of the S-layer proteins encode an aggregation-promoting factor sharing several features with the S-layer proteins (Turpin et al. 2012). Additionally, cell surface–associated cell wall hydrolases Msp2/p40 and Msp1/p75 of *L. casei* have been shown to bind to mucin, collagen, and cultured epithelial cells (Bäuerl et al. 2010).

Comparative studies of lactobacilli have shown that their health efficacy is not only species-specific, but also varies between strains of a species. This observation justifies the need to characterize *Lactobacillus* strains individually, also at the genome level (Ventura et al. 2009; FAO/WHO 2002). In this context, the *L. casei* taxonomic group comprised of three related species (*L. casei*, *L. paracasei*, and *L. rhamnosus*) (Felis and Dellaglio 2007) is of great interest as numerous commonly utilized probiotic strains belong there (Kalliomäki et al. 2003; Sykora et al. 2005; Klaenhammer et al. 2008; Almeida et al. 2012; Kankainen et al. 2009; Douillard et al. 2013; Smokvina et al. 2013). Despite the groups’ importance and apparently comprehensive studies, some species affiliations of their members are turned out to be erroneous, e.g., a strain previously assigned as *L. casei* LOCK 0919 (Koryszewska-Baginska et al. 2013) basing on a re-examination of its genome sequence has now been renamed *L. paracasei* LOCK 0919.

In this study, we isolated a new strain *L. paracasei* subsp. *paracasei* IBB3423 from samples of raw cow milk from northeastern Poland. Functional tests revealed their high adhesion capacity and ability to metabolize inulin. Its DNA was sequenced and predicted to comprise two plasmids in addition to the chromosome. Bioinformatic analyses revealed the presence of a number of genes coding for potential adhesion proteins, including pilus-encoding *spaCBA* genes residing in one of the plasmids. The plasmid’s removal deprived the bacteria of their capacity to adhere, indicating that it most likely depends on the SpaCBA pili.

## Materials and methods

### Isolation of strains, growth conditions, and DNA extraction for genome sequencing

The raw cow milk samples were collected from northeastern Poland and *L. paracasei* subsp. *paracasei* IBB3423 strain was isolated from them by students (Supplementary Information S6) within the citizen science project (“Paths of Copernicus” MakeTogether program). Four other *Lactobacillus* strains (*L. rhamnosus* GG, *L. rhamnosus* LOCK 0900, *L. rhamnosus* LOCK 0908, and *L. paracasei* LOCK 0919) of human origin were obtained from commercial dietary supplements under the brand names of Dicoflor (Bayer, Germany; GG) and Latopic (Biomed, Poland; LOCK 0900, LOCK 0908 and LOCK 0919). Strain IBB3423 was isolated by serial dilution on MRS broth (De Man, Rogosa and Sharpe; Difco) solidified with 1% agar and incubating anaerobically (37 °C, 48 h). Individual colonies were inoculated into liquid MRS and propagated overnight anaerobically at 37 °C. Total DNA was extracted using a Genomic Mini kit (A&A Biotechnology, Poland) according to the manufacturer’s instruction with the following modifications. An overnight culture of *L. paracasei* subsp. *paracasei* IBB3423 in MRS medium was centrifuged (14,000 rpm, 1 min) and the pellet was suspended in TES (0.2 M Tris-HCl pH 8.0, 0.5 mM EDTA, 0.5 M sucrose) with lysozyme (10 mg/ml) and mutanolysin (10 U/ml) and incubated (37 °C, 1 h) with occasional mixing. The suspension was centrifuged (14,000 rpm, 3 min) and the pellet was gently suspended in the supplied buffer. The obtained DNA was characterized and quantified by measuring its OD at 230, 260, and 280 nm. Identification at species level was based on 16S rDNA sequencing following amplification with universal primers 27F (5′-AGAGTTTGATCCTGGCTCAG-3′) and 1492R (5′-GGTTACCTTGTTACGACTT-3′) (Lane 1991). The nucleotide sequence of the PCR product was compared with other sequences available using the Basic Local Alignment Search Tool (BLAST; https://blast.ncbi.nlm.nih.gov/Blast.cgi/; Altschul et al. 1990) and the ANI (average nucleotide identity) calculator software (https://www.ezbiocloud.net/tools/ani/; Yoon et al. 2017), which indicated that IBB3423 belongs to *L. paracasei* subsp. *paracasei* and has the highest similarity to *L. paracasei* subsp. *paracasei* TMW 1.1434.

### Aggregation, hydrophobicity, and adherence tests

The tests for aggregation, hydrophobicity, and adherence to abiotic (glass and polystyrene) and biotic (collagen, gelatine, and mucus) surfaces were performed as described previously (Aleksandrzak-Piekarczyk et al. 2016). The following criteria were used to classify the adherence properties: A ≥ 3, strongly adherent; 3 > A > 2, moderately adherent; 2 > A > 1, weakly adherent; A ≤ 1, non-adherent.

For adherence tests to epithelial tissue, the colorectal carcinoma cell line Caco-2 (Cell Line Service GmbH, Germany) from 42nd passage was cultured in T75 Roux bottles (Greiner Bio-One GmbH, Germany) as described previously (Nowak et al. 2016) in DMEM (Dulbecco’s Modified Eagle’s Medium; Sigma-Aldrich, USA) supplemented with 10% FBS (fetal bovine serum; Thermo Fisher Scientific, USA), 25 mM HEPES (4-(2-hydroxyethyl)-1-piperazineethanesulfonic acid; Sigma-Aldrich, USA), 4 mM GlutaMAX (Thermo Fisher Scientific, USA), a mixture of 100 μg/ml streptomycin, and 100 IU/ml penicillin (Sigma-Aldrich, USA). Cells were incubated (37 °C, 5% CO_2_, humidity > 95%) for 7 days to reach confluence and then were detached with Tryple^TM^ Express (Thermo Fisher Scientific, USA) according to manufacturer’s instruction. The cell suspension was centrifuged (200×*g*, 5 min), the pellet was suspended in fresh DMEM, and the number and viability of cells were determined by trypan blue staining (Sigma-Aldrich, USA). For the adherence assay, Caco-2 cells were placed in a 24-well plate (2.5 × 10^5^ cells/well) and left overnight, while bacteria were cultivated in MRS broth (24 h, 37 °C), centrifuged (9300×*g*, 10 min), washed with phosphate-buffered saline (PBS), and suspended in DMEM without supplements in the amount of 7–8 × 10^8^ CFU/ml. Medium was aspirated from Caco-2 cells and 1 ml of bacteria in DMEM was added. The plate was incubated (2 h, 37 °C, 5% CO_2_, humidity > 95%) and non-adherent bacteria were removed by gentle washing with PBS. Caco-2 cells with adhered bacteria were detached with 200 μl 1% trypsin (Sigma-Aldrich, USA) for 10 min at 37 °C, scraped with a cell scraper (Greiner Bio-One GmbH, Germany), transferred into Eppendorf tubes, and centrifuged (9300×*g*, 10 min). To lyse the Caco-2 cells, the pellet was resuspended in 1 ml 0.1% Triton X-100 (Sigma-Aldrich, USA) and incubated for 5 min at RT. The released bacteria were counted by plating on MRS agar and incubating for 48 h at 37 °C. The adherence rate was calculated as follows: A[%] = (logA2/logA1) × 100, where A1 is the number of colony-forming units (CFU) of initial bacteria added to the well and A2 is the number (CFU) of adhered bacteria.

For microscopic visualization of adhering bacteria, the procedure described above was carried out in an 8-chamber Lab-Tek^TM^ microplate (Thermo Fisher Scientific, USA). After 2 h of incubation, non-adherent bacteria were removed; wells were washed with PBS, fixed with 70% methanol for 15 min, and then stained for 10 min with 0.1% crystal violet. After that, wells were washed with 70% ethanol and dried overnight. Samples were observed at 100× objective under a microscope (Nikon Eclipse Ci H600L, Japan) connected to a Nicon Digital Sight DS-U3 camera using NIS-elements BR 3.0 imaging software (Nikon, Japan).

Data were analyzed using two-way analysis of variance (ANOVA), and differences between values with normal distribution were evaluated by Student’s *t* test using OriginPro 6.1 software (OriginLab Corporation, Northampton, USA). The differences were deemed significant at *P* < 0.05. The results are presented as mean ± standard deviation (SD).

### Genome sequencing and bioinformatic analyses

The total DNA of *L. paracasei* subsp. *paracasei* IBB3423 was subjected to whole genome sequencing (WGS) on a MiSeq system (Illumina, San Diego, CA) at the DNA Sequencing and Oligonucleotide Synthesis Laboratory, IBB PAS. DNA was sheared to the appropriate size and used for paired-end TruSeq (Illumina, USA) library construction following the manufacturer’s instructions. Additionally, the long-span mate-pair library was constructed from non-sheared DNA using Nextera Mate Pair Kit (Illumina, USA) following manufacturer’s instructions. Both libraries were sequenced in the paired-end mode using a v3 (600 cycles) chemistry kit (Illumina, USA). Obtained sequence reads were filtered by the quality and assembled using Newbler v3.0 software (Roche, USA). Gaps remaining in the genome assembly were closed by PCR amplification of DNA fragments followed by Sanger sequencing on an ABI3730xl Genetic Analyzer (Life Technologies, USA) using BigDye Terminator Mix v. 3.1 chemistry (Life Technologies, USA). To obtain complete nucleotide sequence of the genome errors, misassemblies were corrected using the Seqman software (DNAStar, USA). Annotation of open reading frames (*orf*s) and non-coding RNAs was done using the RAST server (http://rast.nmpdr.org/; Aziz et al. 2008) and checked by BLAST analysis when needed. RAST and BASYs (https://www.basys.ca/; Van Domselaar et al. 2005) software were applied for constructing Clusters of Orthologous Groups (COGs) of predicted proteins. Carbohydrate metabolic pathways were reconstructed using BlastKOALA tool from KEGG (Kyoto Encyclopedia of Genes and Genomes; https://www.kegg.jp/blastkoala/; Kanehisa et al. 2016). Carbohydrate-specific enzymes were annotated by the dbCAN prediction web server (http://csbl.bmb.uga.edu/dbCAN/; Yin et al. 2012). Transporters were predicted by searching the TCDB database (http://www.tcdb.org/; Saier et al. 2016) with the BLASTP program with the Expect value (*E* value) lower than e^−05^. Possible bacteriophage sequences were searched using PHAST (http://phast.wishartlab.com/; Zhou et al. 2011). CRISPR loci were identified using the CRISPRFinder tool (http://crispr.i2bc.paris-saclay.fr/; Grissa et al. 2007). Genome visualization was done using CGView server (http://cgview.ca/; Grant and Stothard 2008). The similarity of IBB3423 plasmids to other plasmids was evaluated using the standard BLASTN program at the NCBI site and best hits were used to show sequence similarity using Circoletto with the selected *E* value of 0.1 (http://tools.bat.infspire.org/circoletto/; Darzentas 2010). For comparative studies, the following 25 complete genome sequences of *L. casei* group were downloaded from NCBI: *L. paracasei* ATCC 334 (NC_008526.1), *L. paracasei* FAM18149 (NZ_CP017261.1), *L. paracasei* TK1501 (NZ_CP017716.1), *L. paracasei* IIA (NZ_CP014985.1), *L. paracasei* subsp. *paracasei* TMW 1.1434 (NZ_CP016355.1), *L. paracasei* subsp. *paracasei* 8700:2 (NC_022112.1), *L. paracasei* N1115 (NZ_CP007122.1), *L. paracasei* LOCK 0919 (NC_021721.1), *L. paracasei* subsp. *paracasei* JCM 8130 (NZ_AP012541.1), *L. paracasei* CAUH35 (NZ_CP012187.1), *L. paracasei* L9 (NZ_CP012148.1), *L. paracasei* KL1 (NZ_CP013921.1), *L. paracasei* HD1.7 (NZ_CP025582.1), *L. paracasei* EG9 (NZ_CP029546.1), *L. paracasei* Lpc10 (NZ_CP029686.1), *L. paracasei* LC355 (CP029536.1), *L. paracasei* Zhang (NC_014334.1), *L. paracasei* BD-II (NC_017474.1), *L. paracasei* LC2W (NC_017473.1), *L. paracasei* HDS-01 (NZ_CP026097.1), *L. casei* BL23 (NC_010999.1), *L. casei* 12A (NZ_CP006690.1), *L. casei* W56 (NC_018641.1), *L. casei* LC5 (NZ_CP017065.1), and *L. casei* ATCC 393 (NZ_AP012544.1). The average nucleotide identity between IBB3423 and respective *Lactobacillus* genomes was calculated with the ANI calculator software. Comparisons between chromosomes were performed using the LASTZ program with the step length of 20 and seed pattern of 12 of 19 (https://www.geneious.com/plugins/lastz-plugin/; Harris 2007).

### Data access

The finished genome sequence for *L. paracasei* subsp. *paracasei* IBB3423 comprising the chromosome and two plasmids pLCAKO.1 and pLCAKO.2 has been deposited at the NCBI GenBank database with accession numbers CP022954, CP022955, and CP022956, respectively. The IBB3423 strain has been deposited at the publicly accessible Polish Collection of Microorganisms (PCM), culture no. 3007.

### Sugar fermentation pattern

To determine the sugar fermentation profile of *L. paracasei* subsp. *paracasei* IBB3423 and other *Lactobacillus* strains, the API 50CH kit (BioMerieux, France) was used according to the manufacturer’s instructions. The fermentation patterns were recorded after 48 h of aerobic incubation at 37 °C. Fermentation of carbohydrate was detected by acid production demonstrated by a change in color of the pH indicator present in the medium.

### Plasmid curing

The pLCAKO.2 plasmid was removed from strain IBB3423 by serial passages at a 10^−3^-fold dilution in MRS broth and culturing for 96 h at 37 °C under anaerobic condition. Appropriate dilutions of selected passages (after 4, 12, 20, 28, 32, 40, and 48 days of cultivation) were plated on MRS agar plates. After 48 h of incubation, visible colonies were randomly selected for further analysis. The absence of pLCAKO.2 plasmid was assessed by PCR with primers pLCAKO_3F (5′-CCTCCTTTAGACGCTGAACG-3′), pLCAKO_3R (5′-GGGCGGTACTTTATGGCAAC-3′), pLCAKO_7F (5′-CGCCTATCAAGTCGAAGGAG-3′), and pLCAKO_7R (5′-TCGAGCATCGCCTGCATACG-3′). A strain devoid of pLCAKO.2 was selected after 48 days of cultivation and named IBB3423 ΔpLCAKO.2.

## Results

### Sugar fermentation profile and preliminary studies of adhesion to bare PS plates of *L. paracasei* subsp. *paracasei* IBB3423

The ability of *L. paracasei* subsp. *paracasei* IBB3423 to utilize certain carbohydrates was compared with the fermentation profiles of well-characterized *L. casei* group bacteria with fully sequenced genomes as follows: *L. rhamnosus* GG, *L. rhamnosus* LOCK 0900, *L. rhamnosus* LOCK 0908, and *L. paracasei* LOCK 0919 (Kankainen et al. 2009; Aleksandrzak-Piekarczyk et al. 2013; Koryszewska-Baginska et al. 2013; Koryszewska-Baginska et al. 2014), originally isolated from the human gastrointestinal tract.

**Fig. 1:**
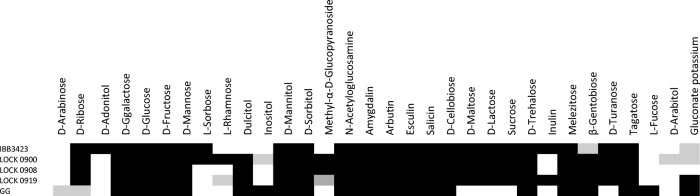
Fermentative profile of *L. paracasei* subsp. *paracasei* IBB3423. A positive result is indicated by black rectangles, partially by gray and negative by white rectangles. No strain could use glycerol, erythritol, d-xylose, l-xylose, methyl-β-d-xylopyranoside, methyl-α-d-mannopyranoside, d-melibiose, d-raffinose, starch, glycogen, d-lyxose, xylitol, d-fucose, l-arabitol, 2-ketogluconate potassium, or 5-ketogluconate potassium

All the strains fermented numerous simple and complex carbohydrates (Fig. 1), but IBB3423 was unique among them being capable of metabolizing inulin, d-adonitol (also called d-ribitol), and l-sorbose but not l-rhamnose, dulcitol, inositol, and l-fucose. The inability to use those sugars is probably related to their low abundance in cow milk.

In addition, *L. rhamnosus* GG and *L. paracasei* LOCK 0919 are strains of a considerable adhesion capacity (Aleksandrzak-Piekarczyk et al. 2016; Segers and Lebeer 2014) in contrast to poorly adherent *L. paracasei* LOCK 0900 and *L. rhamnosus* LOCK 0908 (Aleksandrzak-Piekarczyk et al. 2013; Koryszewska-Baginska et al. 2014). The properties of adhesion to microtiter PS plates of IBB3423, LOCK 0900, LOCK 0908, LOCK 0919, and GG were compared. *L. rhamnosus* GG isolate and *L. paracasei* LOCK 0919 showed high/moderate-level PS binding, while the *L. paracasei* LOCK 0900 and *L. rhamnosus* LOCK 0908 strains showed virtually no binding under the conditions tested. In comparison with them, *L. paracasei* IBB3423 presented the strongest adherences ability, even as much as 35% higher than the best adhesive *L. rhamnosus* GG strain (Figure S1).

### Characteristics of *L. paracasei* subsp. *paracasei* IBB3423 genome

**Fig. 2:**
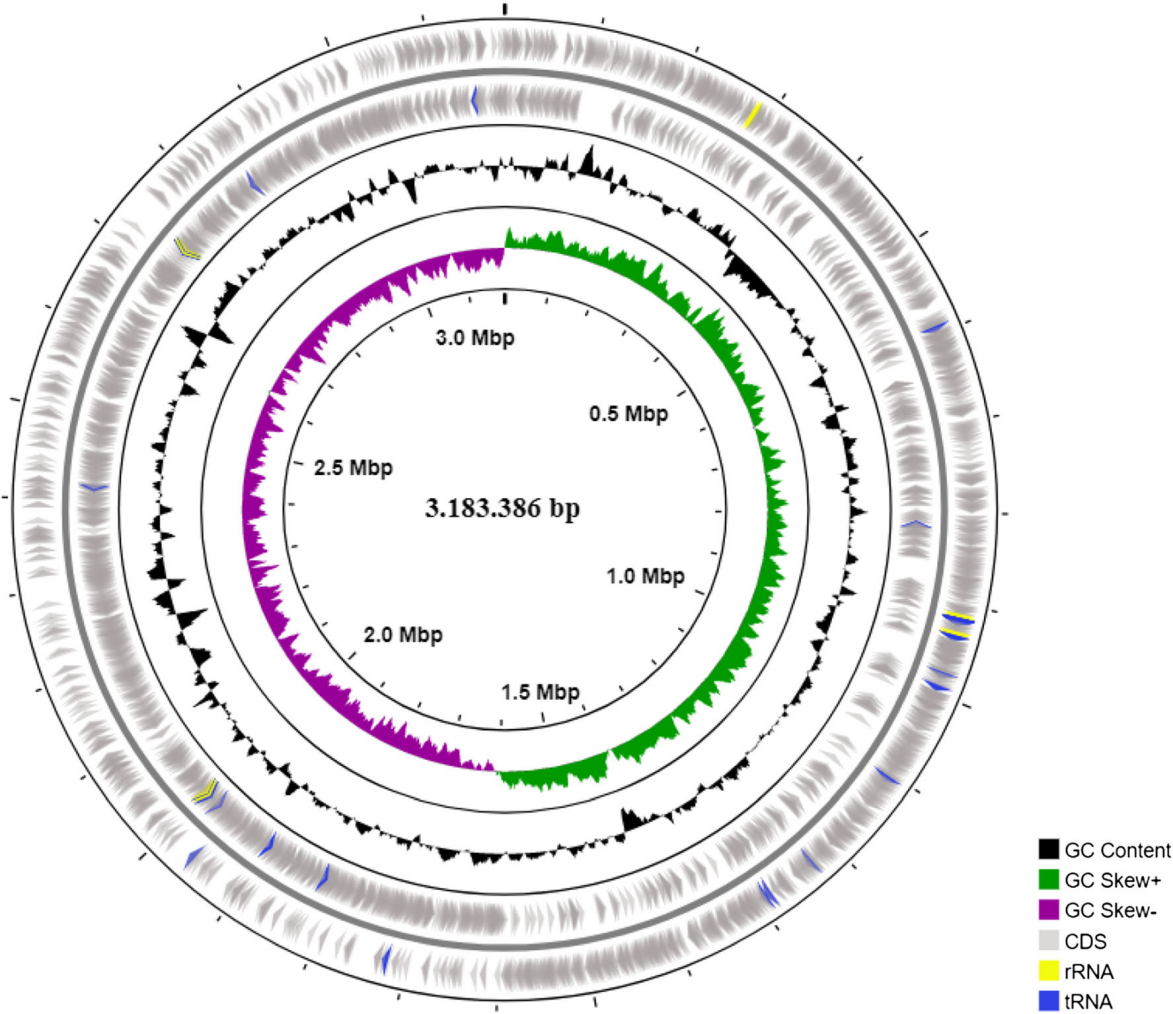
Circular map of *L. paracasei* subsp. *paracasei* IBB3423 chromosome. The first two outer circles indicate predicted genes on forward and reverse strand. rRNA and tRNA genes are depicted by yellow and blue arrows. The black circle represents GC content and the green/purple circle shows GC skew

In order to identify the genetic determinants responsible for the strong adherence of *L. paracasei* subsp. *paracasei* IBB3423 and the wide range of metabolized carbon sources, we sequenced its entire genome. In addition to the circular chromosome of 3,183,386 bp, two circular plasmids designated pLCAKO.1 (5986 bp) and pLCAKO.2 (51,211 bp) were found. The GC content of the chromosome is 46.3% and, respectively, 42.7% and 43.8% for pLCAKO.1 and pLCAKO.2. These values agree well with the GC content in other lactobacilli (Sun et al. 2015); however, local GC content variation in the chromosome and a clear-cut GC skew at the origin of replication is evident (Fig. 2). A total of 3216 genes were identified in the chromosome, of which 3116 were annotated as protein-coding genes, resulting in the coding capacity of 84.5%. As with the GC content, the distribution of genes on the two DNA strands is substantially skewed around *ori* so that their transcription tends to agree with the direction of replicative fork migration. Fifty-nine tRNA genes representing all amino acids were found, and five complete rRNA operons, three on the forward strand, and two on the reverse strand. Most of the tRNAs genes are clustered in the close neighborhood of rRNA locus (Fig. 2). All the rRNA operons and nearly all tRNA genes are arranged in the direction of fork migration. Seventy-six genes were identified on pLCAKO.1 and pLCAKO.2 plasmids (Table 1).

**Table 1:** Characteristics of *L. paracasei* subsp. *paracasei* IBB3423 genome

Chromosome	
Length (bp)	3,183,386
GC content (%)	46.3
Total genes	3216
Coding DNA (%)	85.8
Protein-coding genes	3116
Protein coding capacity (%)	84.5
Genes assigned to COGs	1972
rRNA genes	15
tRNA genes	59
Prophages	3
CRISPR locus	1
Plasmid pLCAKO.1	
Length (bp)	5986
GC content (%)	42.7
Total genes	9
Protein-coding genes	9
Genes assigned to COGs	0
Plasmid pLCAKO.2	
Length (bp)	51,211
GC content (%)	43.8
Total genes	67
Protein-coding genes	64
Genes assigned to COGs	27

A total of 2171 genes from entire genome (~ 67%) could be assigned a putative biological function, while the remaining ~ 33% genes were annotated as hypothetical or of unknown function. When their biological roles according to COG categories were analyzed, out of the total of 3189 predicted proteins, 1999 (62.7%) could be assigned to a COG functional category. About 10% of the proteins are involved in cellular processes and signaling (categories D, M, N, O, T, U, V), 16% in information storage and processing (categories J, K, L), almost 26% in metabolism (categories C, E, F, G, H, I, P, and Q), and ca. 11% are poorly characterized (categories R and S) (Supplementary Table S1). Proteins related to carbohydrate and amino acid transport and metabolism are particularly numerous, consistent with the wide fermentation profile of the strain and the inability of lactobacilli to synthetize most amino acids. Notably, no genes associated with pathogenesis were found, indicating that the IBB3423 strain should be safe to use as a probiotic.

A widespread abundance of prophages in the genomes of *Lactobacillus* spp. has been reported (Mercanti et al. 2016) and IBB3423 was typical in this respect. Four intact prophage regions ranging in length from 24.9 to 41.5 kb partially homologous to prophage sequences widely distributed among other lactobacilli were identified. All of them contain a phage attachment (ATT) site. Judging from the presence of genes involved in bacterial lysis (holin and phage lysin), two of those phages may follow the lysogenic pathway.

Clustered regularly interspaced short palindromic repeats (CRISPRs) are components of the type of bacterial immune system against mobile genetic elements, which are often found adjacent to the *cas* gene cluster encoding Cas protein complex (Haft et al. 2005). Type II-A/Lsal1 CRISPR-*cas* system is present in the IBB3423 genome. The CRISPR locus contains 26 perfect repeats of a 36-bp-long sequence (5′GCTCTTGAACTGATTGATTCGACATCTACCTGAGAC) and one imperfect repeat (5′ACTCTTGAACTGATTGATTCGACATCTACCTGAGAC). Four CRISPR-associated *cas* genes (*LCAKO_2393*, *LCAKO_2394*, *LCAKO_2395*, and *LCAKO_2396*) are present upstream of the DNA repeats.

**Fig. 3:**
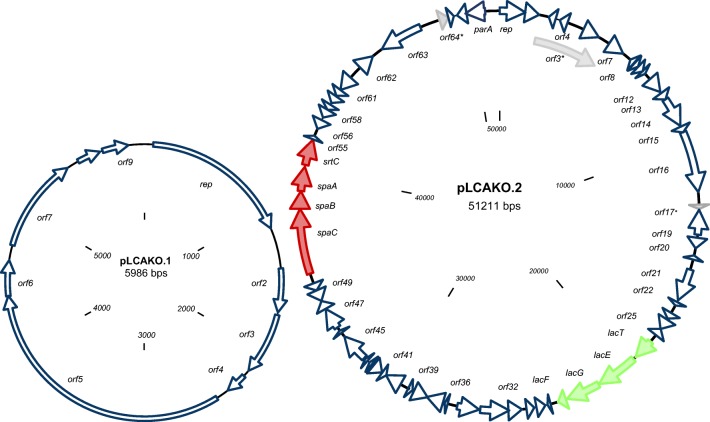
Circular genetic maps of plasmids pLCAKO.1 and pLCAKO.2 of *L. paracasei* subsp. *paracasei* IBB3423. *orf*s are indicated by arrows showing the direction of transcription*.* The pilus-encoding *spaCBA-srtC* cluster is colored in red and lactose operon in green; an asterisk indicates pseudogenes. The two maps are drawn not to scale

The sequence assembly of the entire IBB3423 genome clearly indicated the presence of two plasmids: pLCAKO.1 and pLCAKO.2. Their organization is shown in Fig. 3. The smaller pLCAKO.1 plasmid contains nine genes, all transcribed in the clockwise orientation and mostly encoding proteins with unknown or hypothetical functions homologous to proteins from non-lactobacilli (Supplementary Table S2). The product of the *repB* gene harbors a Rep_trans superfamily domain (pfam02486), which is characteristic for rolling circle replicating (RC) plasmids (Balson and Shaw 1990).

The lager pLCAKO.2 harbors 67 putative genes, of which 64 were predicted to code for proteins. Most exhibit homology with genes located on chromosomes or plasmids of other lactobacilli. Putative biological functions could be assigned to 35 proteins. Fifteen transposase/insertion sequences and one gene encoding pin-related site recombinase/DNA invertase are present in pLCAKO.2 (Supplementary Table S2). The abundance of such elements likely reflects the occurrence of numerous rearrangements during plasmid evolution. The RepA protein encoded by pLCAKO.2 (gene *LCAKO_2p1*) contains RepA_N domain (pfam06970), which is characteristic for class F family pL32-type theta-replication proteins (Tanaka and Ogura 1998). That pLCAKO.2 is a theta-replicating plasmid is additionally indicated by its substantial size (~ 50 kb) as it has been shown that plasmids of this size found in natural isolates of *Lactobacillus* have been shown typically to replicate via the theta mechanism (Wang and Lee 1997). Plasmid replication and stability are closely related, and downstream of *repA* lies *orf67* encoding a protein assigned to the ParA family of ATPases; they are involved in segregational stability preventing plasmid loss during cell division.

Among the genes encoding proteins of known function, pLCAKO.2 harbors genes encoding serine acetyltransferase, cystathionine gamma-lyase, and cystathionine beta-synthase enzymes, which are involved in amino acid metabolism and a lactose-specific PTS operon (*Lac*-PTS) responsible for lactose uptake and fermentation, comprising *lacTEGF* genes (*LCAKO_2p26*–*LCAKO_2p29*), and is absent in the chromosome. No such operon is present in the chromosome. Noteworthy is another operon with known function, the pilus- and sortase-encoding *spaCBA-srtC* gene cluster (*LCAKO_2p51*–*LCAKO_2p54*), which could play an important role in the adhesion to host cells. Its presence prompted us to investigate the dependence of the excellent adherence of IBB3423 on the pLCAKO.2 plasmid.

**Fig. 4:**
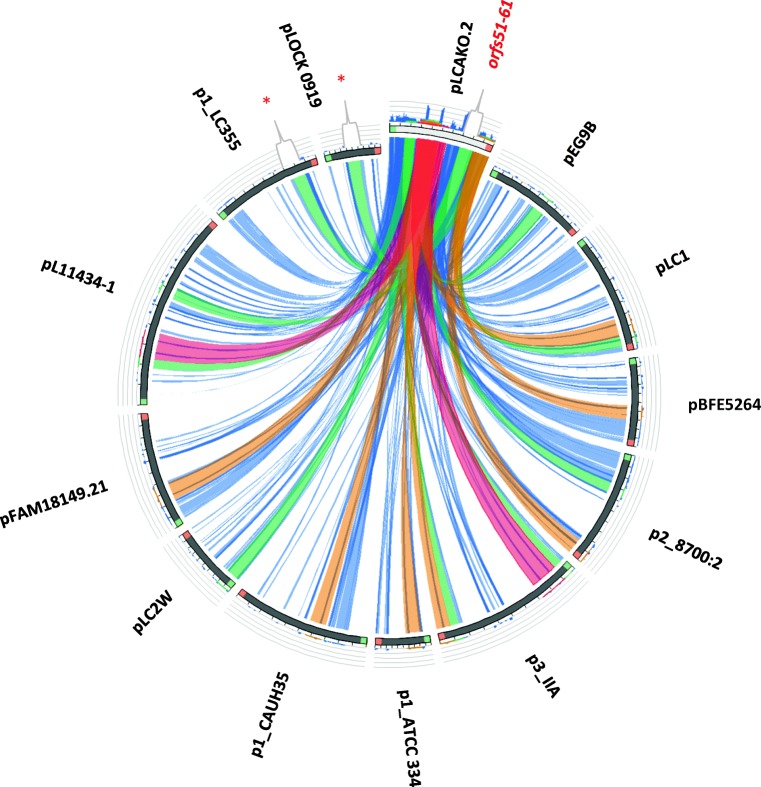
Sequence similarity between pLCAKO.2 plasmid and other lactobacilli plasmids. Plasmid sequences are placed at the circumference. Ribbons represent local alignments produced by BLAST, their width shows alignment length, and colors blue, green, orange, and red correspond to the alignment bit scores in the four quartiles, from the lowest (bit score below 25%) to the highest (bit score between 75 and 100%). Twisted ribbons connect sequences in opposite orientations (inverted). An asterisk indicates region homologous to the *spaCBA-srtC* cluster and downstream genes (*orfs51–61*) from the pLCAKO.2 plasmid. The following plasmid sequences were compared: pL11434-1 GenBank accession no. NZ_CP016356.1 from *L. paracasei* subsp. *paracasei* strain TMW 1.1434; pFAM18149.21 NZ_CP017262.1 from *L. paracasei* FAM18149; p3_IIA NZ_CP014988.1 from *L. paracasei*; p1_ATCC 334 NC_008502.1 from *L. paracasei* ATCC 334; p1_CAUH35 NZ_CP012188.1 from *L. paracasei* CAUH35; p2_8700:2 NC_022123.1 from *L. paracasei* subsp. *paracasei* 8700:2; p1_LC355 NZ_CP029537.1 from *L. paracasei* LC355; pEG9B NZ_CP029548.1 from *L. paracasei* EG9; pLOCK 0919 NC_021722.1 from *L. paracasei* LOCK 0919; pLC2W NC_017475.1 from *L. paracasei* LC2W; pLC1 NC_013200.1 from *L. rhamnosus* Lc 705; pBFE5264 NZ_CP014202.1 from *L. rhamnosus* BFE5264

pLCAKO.1 shows no significant homologies with other plasmids deposited in the GenBank. In contrast, pLCAKO.2 exhibits conservation of almost all its sequence with other plasmids, albeit its gene layout is unique. This unique architecture confirms the earlier suggestion of numerous rearrangements based on the high content of mobile genetic elements in IBB3423. The most similar plasmid is pL11434-1 from *L. paracasei* subsp. *paracasei* TMW 1.1434 along 76% of the pLCAKO.2 length, including mobile elements and genes encoding *Lac*-PTS, serine acetyltransferase, cystathionine lyase, and several hypothetical proteins. Notably, a region homologous to the *spaCBA-srtC* cluster and downstream genes (*orfs51–61*) is present in two plasmids only—pLOCK 0919 (Aleksandrzak-Piekarczyk et al. 2016) and p1_LC355 (NZ_CP029537.1) (Fig. 4).

Bearing in mind the unusually wide range of carbohydrates assimilated by IBB3423, we searched its genome factors involved in sugar metabolism. As expected, proteins assigned to the carbohydrate transport and metabolism category were many and constituted over 8% of all predicted proteins (Supplementary Table S1). The majority of carbohydrate transporters found in bacteria represents phosphoenolpyruvate (PEP)-dependent phosphotransferase systems (PTSs). Thirty-nine genes encoding PTS permeases from all seven recognized families, with various sugar substrate specificities are present in the IBB3423 genome. In addition, there are several transporters from the ATP-binding cassette (ABC) family and secondary sugar transporters from the major facilitator superfamily (MFS) (Supplementary Table S3). Many of these genes lie close genes encoding a glycosidase and transcriptional regulator, allowing hydrolysis of the incoming sugar and local transcriptional control. More than 40 glycosidases found include among others, 6-phospho-β-glucosidase (six genes), β-glucosidase (one gene), 6-phospho-β-galactosidase (one gene), β-galactosidase (two genes), *α*-glucosidase (four genes), *α*-galactosidase (three genes), *α*-fucosidase (two genes), and neopullulanase (two genes). Notably, the LCAKO_0451 glycosidase with a predicted β-fructosidase specificity contains a canonical LPXTG signal sequence, which suggests its extracytoplasmic localization. We posit that this enzyme could be important due to the strain’s ability to utilize the polyfructan inulin. Due to its size, inulin cannot enter the bacterial cell and has to be fragmented by an extracellular activity first to be used as a carbon source. Other genes encoding various carbohydrate-modifying enzymes from several CAZy “carbohydrate-active enzymes” families comprised numerous glycosyltransferases (39 genes), carbohydrate esterases (16 genes), carbohydrate-binding modules (5 genes), and one polysaccharide lyase. Additionally, members of the predicted carbon catabolite regulation network were identified, namely catabolite control protein A (CcpA; LCAKO_0880), phosphoryl transfer enzyme I (EI) (LCAKO_1965), HPr (LCAKO_1964), and HPr kinase/phosphorylase (LCAKO_1110).

The capacity to metabolize complex carbohydrates such as inulin is reflected in the IBB3423 genome. A putative operon involved in inulin degradation homologous to the *fos* cluster from other *L. paracasei* strains was identified (Goh et al. 2006). The *fosRABCDXE* operon of IBB3423 comprises genes encoding a transcriptional regulator FosR (LCAKO_0445), components of a putative fructose/mannose-specific PTS (LCAKO_0446–LCAKO_0450), and a β-fructosidase precursor FosE (LCAKO_0451). An intact glycogen metabolic pathway encoded by the *glgBCDAP-amyB* genes (*LCAKO_2211* to *LCAKO_2216*) organized identically to the corresponding operons of other *Lactobacillus* species (Goh and Klaenhammer 2014) was identified in IBB3423.

In addition to the above, 15 common carbohydrate utilization pathways were predicted in the IBB3423 genome including glycolysis/gluconeogenesis, the citrate cycle, the pentose phosphate pathway, fructose, mannose, galactose, ascorbate, glucuronate, aldarate, sucrose, amino sugars and nucleotide sugars, pyruvate, glyoxylate and dicarboxylate, propanoate, butanoate metabolism, C5-branched dibasic acids, and inositol phosphates. In addition to the gene complement for glycolysis, six lactate dehydrogenase genes (both d- and l-) for the conversion of pyruvate into d- and l-lactate were identified.

The *L. paracasei* subsp. *paracasei* IBB3423 genome also contains a number of genes potentially involved in the biosynthesis of exopolysaccharides (Schmid et al. 2015) such as two of 28 kb gene clusters (*LCAKO_2172* to *LCAKO_2293*) and 20 kb (*LCAKO_2225* to *LCAKO_2244*).

### Adhesion properties of strain IBB3423

To determine the adhesive properties of IBB3423, we performed a series assays using *L. rhamnosus* GG and *L. paracasei* LOCK 0919 as positive references (Aleksandrzak-Piekarczyk et al. 2016; Segers and Lebeer 2014) and *L. rhamnosus* LOCK 0908 as negative reference (Koryszewska-Baginska et al. 2014). To verify the role of the *spaCBA-srtC* gene cluster in adhesion, the IBB3423 strain was cured of the pLCAKO.2 plasmid and the adhesion properties of the resulting *L. paracasei* subsp. *paracasei* IBB3423 ΔpLCAKO.2 strain were compared with those of the parental IBB3423 and other control strains.

The hydrophobicity of the five strains testes varied significantly. IBB3423 showed the highest value (17.4% ± 6.8%), while the same strain after removing SpaCBA pili-encoding plasmid (*L. paracasei* subsp. *paracasei* IBB3423 ΔpLCAKO.2) was almost non-hydrophobic (0.1% ± 0.02%) (Table 2) (*P* < 0.05). The hydrophobicity of GG and LOCK 0908 was similar (3–4%) but significantly lower than that IBB3423 while, rather unexpectedly, strain 0919 was at least hydrophobic (ca. 1.5%) (Table 2). In contrast, the aggregation percentage after 24 h for all the strains except *L. rhamnosus* LOCK 0908, which was the least aggregative, was comparable (more than 80%) (Table 2).

**Table 2:** Hydrophobicity and aggregation ability of *Lactobacillus* spp. strains. Data are means from six repeats in two independent experiments (± SD). Results significantly different from *L. paracasei subsp. *paracasei* IBB3423 and **L. rhamnosus LOCK 0908 (ANOVA, *P* < 0.05)

Bacterial strain	Hydrophobicity (%)	Aggregation (%)
*L. paracasei* subsp. *paracasei* IBB3423	17.4 ± 6.8	83.7 ± 12.7**
*L. paracasei* subsp. *paracasei* IBB3423 ΔpLCAKO.2	0.1 ± 0.02*	81.9 ± 12.6**
*L. rhamnosus* GG	3.9 ± 3.2*	84.2 ± 6.4**
*L. paracasei* LOCK 0919	1.5 ± 0.7*	84.8 ± 9.2**
*L. rhamnosus* LOCK 0908	3.3 ± 1.4*	65.9 ± 5.1

**Fig. 5:**
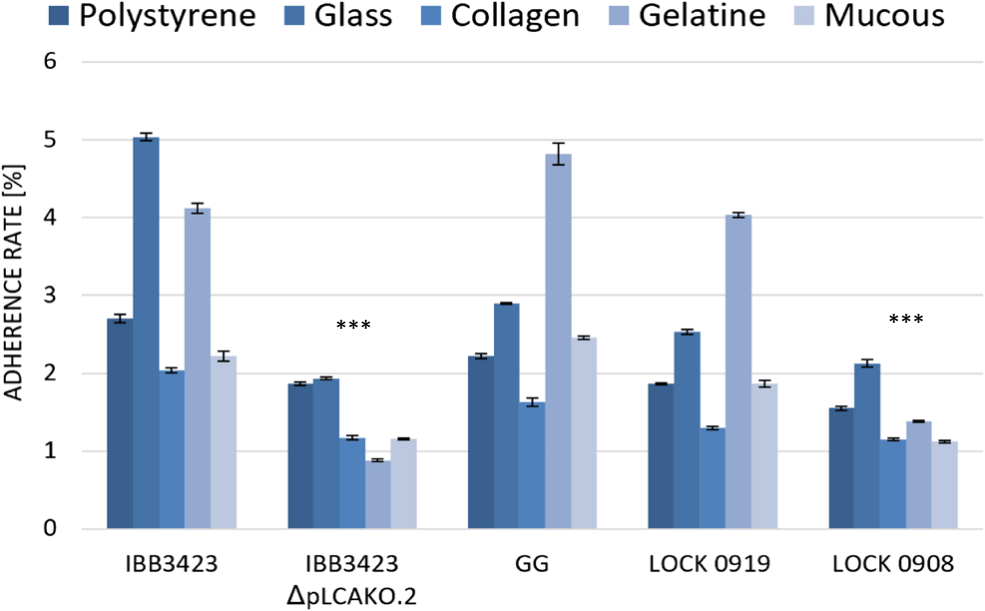
Adherence of *Lactobacillus* strains to abiotic and biotic surfaces. Data represent means from three to eight repeats (± SD). ***Results are significantly different from *L. paracasei* subsp*. paracasei* IBB3423 (ANOVA, *P* < 0.05)

Also the adherence of the bacteria tested to biotic and abiotic surfaces varied, and again, IBB3423 demonstrated the highest adherence ability to all surfaces (*P* < 0.05; Fig. 5). It adhered strongly to glass and gelatine and was medium adhesive to polystyrene, collagen, and mucus. The same strain (IBB3423 ΔpLCAKO.2) after removing its plasmid was significantly less adhesive demonstrating weak adhesion to all surfaces and non-adherence to gelatine. LOCK 0919 and GG showed strong, medium, or weak adhesive properties, depending on the surface, but always adhered better than the negative reference LOCK 0908 (Fig. 5).

**Fig. 6:**
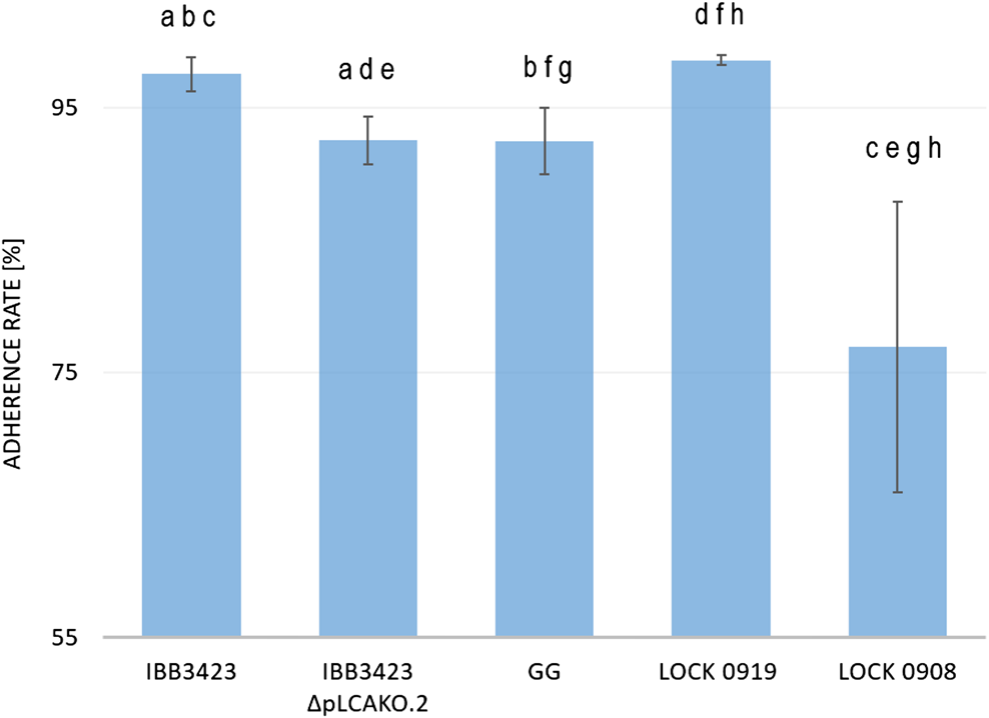
Adherence of *Lactobacillus* strains to Caco-2 cells. Data represent means from three repeats (± SD). ^a–h^Results statistically different (ANOVA, *P* < 0.05).

**Fig. 7:**
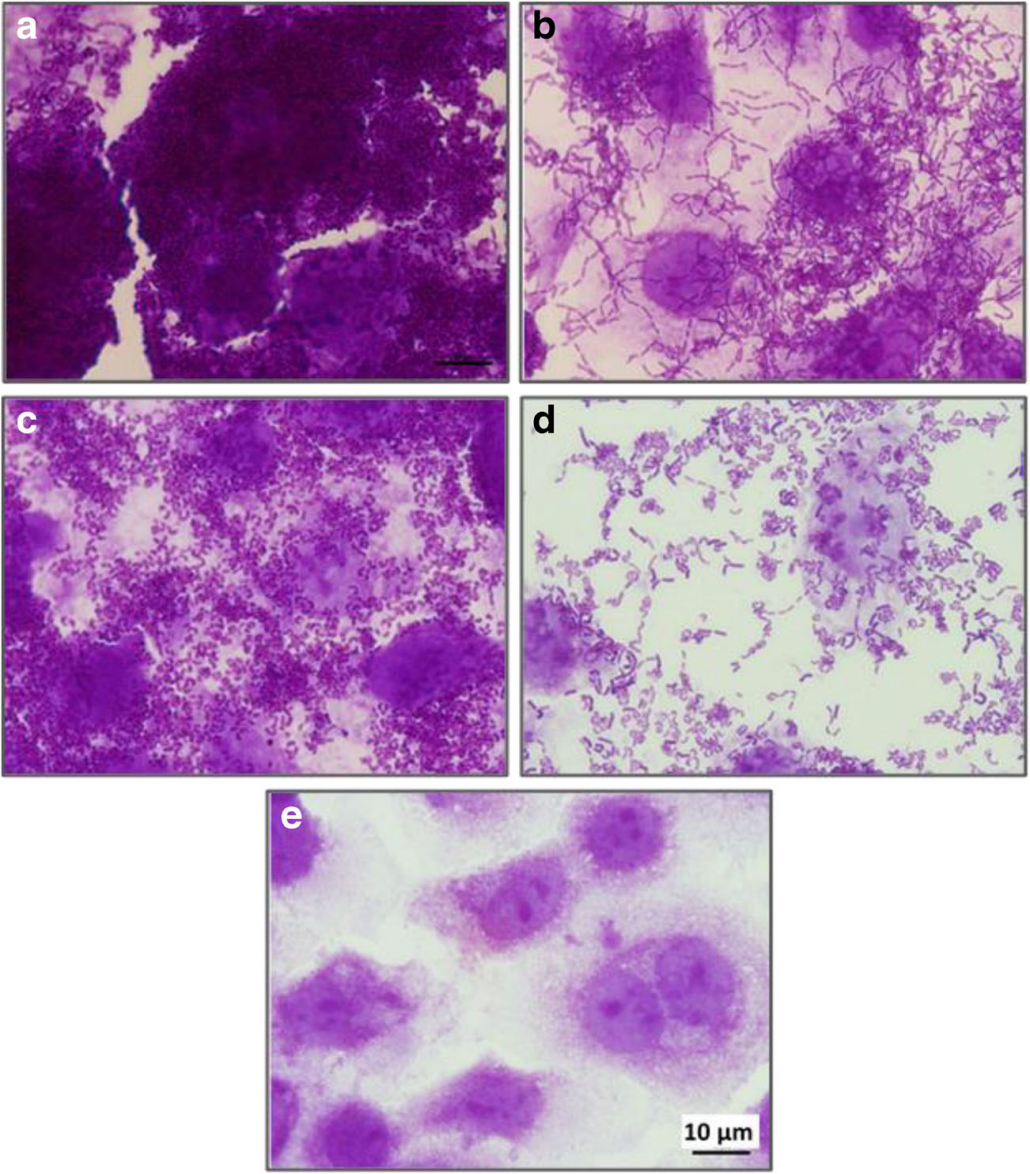
Adherence of various *Lactobacillus* strains to Caco-2 cells. **a***L. paracasei* subsp. *paracasei* IBB3423, **b***L. paracasei* subsp. *paracasei* IBB3423 ΔpLCAKO.2, **c***L. paracasei* LOCK 0919, **d***L. rhamnosus* LOCK 0908, and **e** Caco-2 cells (control). Representative microphotographs after staining with 0.1% crystal violet are shown

To extend those characteristics to an in vivo situation more relevant to the bacteria–host interactions, we used epithelial Caco-2 cells as a model. *L. paracasei* subsp. *paracasei* IBB3423 adhered strongly to Caco-2 cells with an adherence rate of 97.5% ± 1.3%; LOCK 0919 and GG showed a similar lower rate, while *L. rhamnosus* LOCK 0908 displayed much weaker adherence (76.9% ± 10.9%) as shown in Fig. 6, which confirmed the earlier data (Aleksandrzak-Piekarczyk et al. 2016). Also, the elimination of pLCAKO.2 diminished adhesiveness to Caco-2 cells (adherence rate 92.5% ± 1.8%). The high standard deviation for that strain can be explained by the production and excretion of an amorphous, loose, slime-like substance strongly affecting the cell surface characteristics and probably disturbing its adherence (Nowak et al. 2016). Examples of microscopic pictures of adherence of *Lactobacillus* spp. strains to Caco-2 monolayer, confirming stronger adhesive properties of *L. paracasei* subsp. *paracasei* IBB3423 to Caco-2 cells than *L. paracasei* subsp. *paracasei* IBB3423 ΔpLCAKO.2, are presented in Fig. 7.

### Genetic determinants of the adhesion capacity of IBB3423 strain

Cell surface proteins play a critical role in the molecular interactions between bacteria and their host. To understand the reasons for the high adhesive abilities of IBB3423, we searched its genome for factors that could be involved in adhesion to mucus, ECM, or epithelial cells. We identified 54 proteins encoded in the chromosome and five in the pLCAKO.2 plasmid potentially involved in specific adherence mechanisms (Supplementary Table S4), including 22 LPXTG-containing proteins, among which 20 also had a signal sequence for secretion. Among the LPXTG-containing proteins were pilin proteins SpaCBA (chromosomally encoded LCAKO_0529, LCAKO_0530 and LCAKO_0531, and plasmidically encoded pLCAKO.2_51, pLCAKO.2_52, and pLCAKO.2_53) and SpaDEF (LCAKO_2550, LCAKO_2551, and LCAKO_2552), all associated with a dedicated sortase. The SpaCBA proteins encoded by pLCAKO.2 plasmid shared 94%, 88%, and 95% amino acid identity, respectively, with their chromosomally located counterparts. We also detected eight proteins with diverse of collagen-binding domains such as Cna_B, collagenBindB, and collagen_bind and identified six proteins with one or three putative mucin-binding domains (MucBP). Other potential mucin-interacting proteins (LCAKO_0451, LCAKO_0596, and LCAKO_0985) contain BID_2 and Big_3 Ig-like domains. Furthermore, LCAKO_2606, LCAKO_2612, and LCAKO_2619 contain ECM-adhesive ZnuA domains, and LCAKO_1644, a fibronectin-binding protein A domain (FbpA).

We also identified a large surface-associated LCAKO_0110 protein of 3254 amino acids, with repeats of serine, alanine, and aspartic acid (Supplementary Table S4) encoded in a region carrying three glycosyltransferase genes. It has been suggested that glycosyltransferase carry out *O*-linked glycosylation of cell surface proteins on serine residues, thus creating a mucin-like structure (Tettelin 2001).

### Comparative genome analysis of *L. paracasei* subsp. *paracasei* IBB3423

To further characterize IBB3423, we compared its genome with publicly available genomes of *L. casei/paracasei* strains. Complete genomes of five *L. casei* and 20 *L. paracasei* strains were found in the GenBank database, ranging in size between 2.9 and 3.2 Mbp, with a GC content of 46.2–47.9%, and predicted to encode 2600–3300 proteins (Supplementary Table S5). The *L. paracasei* subsp. *paracasei* IBB3423 genome falls within these values and shows > 98% identity with most of these species. The lowest degree of identity (< 80%) was with of *L. casei* LC5 and *L. casei* subsp. *casei* ATCC 393, while the highest (99.7%) with *L. paracasei* subsp. *paracasei* TMW 1.1434.

**Fig. 8:**
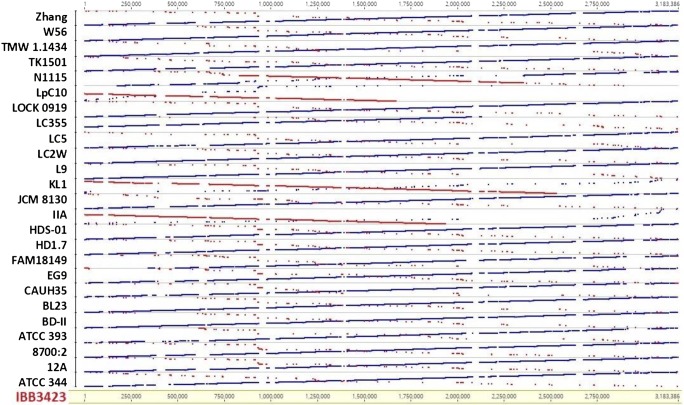
Comparison of chromosome sequence organization between IBB3423 and other completely sequenced *L. casei/L*. *paracasei*. Similar regions in the same and inverted arrangement are shown in blue and red, respectively

A detailed analysis revealed several local differences separating IBB3423 from all the other strains investigated. Thus, the genomes of four *L. paracasei* strains (IIA, KL1, N115, and Lpc10) contained inversions of the long, respective part of the IBB3423 chromosome (Fig. 8). In turn, IBB3423 differed from other strains in gene order in three separate regions (Fig. 8). Another unique feature of IBB3423 is the presence of multiple phage genes scattered across the genome. Several genes of IBB3423 are absent from the genomes from the *L. casei* group; they encode a cell surface protein containing the KxYKxGKxW signal peptide and MucBP and legume-lectin domains (*LCAKO_0636*), a transcription regulator (*LCAKO_0637*), an ATP-dependent endonuclease (*LCAKO_1429*), an AlwI family type II restriction endonuclease (*LCAKO_1430*), a methyl-directed repair DNA adenine methylase (*LCAKO_1431*), the bipolar DNA helicase HerA (*LCAKO_2128*), and a hypothetical protein (*LCAKO_2129*). Other IBB3423 genes are even not found in any other *Lactobacillus* spp., such as *LCAKO_2843* and *LCAKO_2938*, *LCAKO_3065* encoding hypothetical proteins, and *LCAKO_3181* encoding an FRG domain-containing protein.

## Discussion

*Lactobacillus casei/paracasei* strains can be found in various environments and this wide range explains the broad spectrum of their applications in dairy production, biotechnological, and health-related fields (Widyastuti and Rohmatussolihat 2014; Cai et al. 2009; Toh et al. 2013; Douillard et al. 2013; Smokvina et al. 2013). Owing to this potential, they are among the best-explored lactobacilli and new strains from the *L. casei* taxonomic group with novel functional properties are of great interest to both basic science and the biotech industry. In the present study, we isolated from raw cow milk and characterized a novel strain of attractive properties and named it IBB3423. It was recognized as *L. paracasei* based on its 16S rDNA partial sequence (Lane 1991) following current taxonomic guidelines (Judicial Commission of the International Committee on Systematics of Bacteria 2008). Whole genome sequencing confirmed the species identity and indicated the subspecies as *paracasei*. The strain showed desirable characteristics as a potential probiotic strain mainly due to its high adhesion capacity. Naturally, before this strain can get the probiotic status, there is a need to perform other proper tests confirming its probiotic properties and ability to survive in harsh conditions in the digestive tract of animals. According to the FAO/WHO guidelines (FAO/WHO 2002), these tests should include in vitro assays of, e.g., resistance to bile salts and gastric acidity, antimicrobial activity against pathogenic bacteria, the presence of transferable antibiotic resistance genes, and assessment of safety. It is also recommended to validate in vivo probiotic properties of the bacterial strain and to substantiate of health effects in the target host (FAO/WHO 2002).

To date, genomic sequences of 132 *L. casei* and *L. paracasei* strains are publicly available, 25 of which are complete (http://www.ncbi.nlm.nih.gov, last accessed in October 2018). The IBB3423 strain shows genomic features, not unlike those of other *L. casei/paracasei* strains as concerns the GC content (46.3%) and genome size. Notably, its chromosome of ca. 3 Mbp is the second largest among *L. casei/paracasei*. Also, the presence of two plasmids and their size (5986 bp and 51,211 bp) are typical for this group of bacteria. Previous studies have reported that strains of *L. paracasei* harbor up to four (Desmond et al. 2005) or perhaps even six plasmids (Smokvina et al. 2013) of different sizes. However, a recently published article claimed that the *L. paracasei* DPC2071 strain may harbor up to eleven plasmids (Stefanovic and McAuliffe 2018). These mobile replicons often carry genes conferring competitive advantage for the bacterium or critical for the industrial application such as those encoding production of bacteriocins, resistance to antibiotics, heavy metals, and phages or enabling utilization of lactose (Wang and Lee 1997). However, among the plasmid-borne genes of *L. paracasei*, only some have a known function (Smokvina et al. 2013). Also in the present study, many of the predicted proteins encoded by pLCAKO.1 and pLCAKO.2 plasmids were annotated as hypothetical (90% and 45%, respectively). Among those of known function are the pLCAKO.2 genes for lactose metabolism and pilus formation, both features relevant to the possible application of IBB3423. Interestingly, in contrast to pLCAKO.2, the smaller pLCAKO.1 plasmid encodes mainly proteins whose homologs are onlyfound in other genera, such as *Staphylococcus*, *Pseudomonas*, *Enterococcus*, and *Streptococcus*, indicating its possible horizontal transfer from distantly related bacteria.

The genomic relatedness between IBB3423 and other *L. casei/paracasei* strains is the strongest (99.7% identity) with the commercial strain *L. paracasei* subsp. *paracasei* TMW 1.1434. This strain is isogenic with *L. paracasei* subsp. *paracasei* F19 (Schott et al. 2016), a known probiotic strain able to bind gastric human and bovine mucin, collagens I and III, and fibronectin (Di Cerbo and Palmieri 2013). However, an alignment of the two genome sequences (Fig. 8) shows some unique regions in IBB3423. They contain mainly genes for phage-related proteins and hypothetical proteins, but also some of the known predicted functions such as cystathionine gamma-lyase, ABC-type amino acid transporter, type I restriction-modification system subunit R and M, or subunit M of type III R/M system. The overall high identity between IBB3423 and TMW 1.1434 explains to some extent their similar high adhesiveness, although they are of different origins: dairy and gastrointestinal, respectively. One should bear in mind, however, that the isolation source of a strain needs not to be the same as the original niche in which it has evolved, as strains can change habitats due to their adaptability (Ceapa et al. 2015).

The ability to utilize carbohydrates requires the presence of specific transporters for sugar uptake and functional metabolic pathways for its catabolism. In *L. paracasei* subsp. *paracasei* IBB3423, proteins from the functional category of carbohydrate transport and metabolism are the most abundant. This strain encodes enzymes of the Embden–Meyerhof–Parnas (EMP) and phosphoketolase pathways for homolactic and heterolactic lactic acid production from hexoses (Kandler 1983), as well as oxidative and non-oxidative branches of the pentose phosphate pathway (PP) (Tanaka et al. 2002) for utilization of pentoses. However, the non-oxidative PP pathway seems to lack a transaldolase-encoding gene. Regarding the conversion of pyruvate to lactate, at least six d- and l-lactate dehydrogenases are encoded in the IBB3423 genome. The complete Leloir pathway responsible for galactose utilization is also present. In bacteria, carbohydrate uptake is mediated by different classes of transporters, including ABC transporters, secondary transporters (permeases), and PTS transporters (Saier 2000). The PTS, involved in the uptake of mono- and oligosaccharides, is the primary sugar transport system in many lactobacilli (Lorca et al. 2007), but ABC transporters are also important for the transport of oligosaccharides (Monedero et al. 2007). The IBB3423 strain harbors a vast assortment of transporters—at least 39 PTS permeases, several ABC transporter family permeases, and other sugar transporters from the MFS superfamily. Besides these transporters for carbohydrate uptake, the IBB3423 genome encodes numerous carbohydrate-modifying enzymes, which reflects the strain’s sugar fermentation versatility including its unique ability to metabolize inulin, d-adonitol, and l-sorbose. Indeed, all the genes required for sorbose utilization, i.e., those encoding l-sorbose-phosphate-reductase and its transcriptional regulator, sorbitol-6-phosphate dehydrogenase, four components of a sorbose-specific PTS, and fructose-bisphosphate aldolase (Yebra et al. 2000) are present in the IBB3423 genome. Eleven genes for d-adonitol metabolism (*LCAKO_2999* to *LCAKO_3009*) encode components of a mannose-type PTS, a transcriptional regulator, and six other enzymes (Bourand et al. 2013). Also, the *fos* operon involved in utilization of fructo-oligosaccharides (FOS), such as inulin, and the transport of free fructose (Goh et al. 2006) was identified in the IBB3423 genome. As inulin, one of the most studied prebiotics, is known to aid the development of desirable gastrointestinal microflora (Kolida et al. 2002), the ability to metabolize it is a very important property of probiotic bacteria. Among the bacteria that do metabolize prebiotic oligosaccharides are some strains of *Lactobacillus* and *Bifidobacterium* spp., and numerous in vitro and in vivo studies have shown that their growth is stimulated by FOS or other oligosaccharides. On the other hand, comparing with the control strains used by us, isolated from the human gastrointestinal tract, IBB3423 does not have the ability to ferment l-rhamnose, dulcitol, inositol, and l-fucose, probably due to their low abundance in cow milk. Interestingly, an in silico analysis has identified a complete *myo*-inositol (MI) utilization operon identical to that of the probiotic *L. casei* BL23 strain (Yebra et al. 2007), but it appears to be non-functional. Inositol, a sugar alcohol found, among other sources, in soil, is used for phosphate storage in plants, but is rarely used as an energy source by LAB (Yebra et al. 2007). So far, only some strains of *L. casei* have been found to metabolize MI with poor efficiency. One should note that even the presence of genes encoding enzymes for MI metabolism may not be sufficient to confer an ability to utilize MI (Zhang et al. 2010; Vinay-Lara et al. 2014). Moreover, we identified *glgBCDAP-amyB g*enes connected with glycogen metabolism (*LCAKO_2211* to *LCAKO_2216*) organized identically to operons of other *Lactobacillus* species (Goh and Klaenhammer 2014). It remains to be determined why IBB3423 cannot utilize glycogen despite having the relevant genes. Several reasons could be at play, including a lack of expression of these genes or their mutation.

Adherence to the intestinal surface is considered one of the most significant features of probiotic bacteria, as it facilitates colonization of the host and thus persistent protection against pathogens (Jankowska et al. 2008; Lim 2012). Bacteria of the genus *Lactobacillus* are able to adhere to various surfaces, but this feature is highly variable due to the variable presence of genes involved in adhesion. The adherence of *L. paracasei* subsp. *paracasei* IBB3423 to most surfaces is even higher than that of the best adhesive *L. rhamnosus* GG strain. Such high adhesiveness of a dairy strain is unexpected, as strains deriving from milk environments typically display weaker adhesion efficiency than those isolated from intestines/feces (Douillard et al. 2013). The very high adhesive capacity of IBB3423 appears to reflect the presence of as many as 59 genes encoding proteins containing putative adhesion domains, among them are SpaCBA and SpaDEF pilins encoded by chromosomal operons *spaCBA* and *spaDEF* and additional *spaCBA* operon on the pLCAKO.2 plasmid. All these operons had the same gene order and were associated with a gene for a pilin-specific sortase. To date, the SpaCBA proteins have been found mainly in the *L. casei* taxonomic group and much less frequently in other lactobacilli (Aleksandrzak-Piekarczyk et al. 2016). However, despite possessing complete pili operons, some *L. casei* strains do not form pili due to variations in the *spaCBA* sequence (Toh et al. 2013) or its transcriptional incompetence caused by a lack of functional − 35 and − 10 sequences (Aleksandrzak-Piekarczyk et al. 2016). In contrast to *L. casei*, in *L. rhamnosus* GG, the orthologous pili gene cluster is expressed and confers strong mucus-binding ability (Kankainen et al. 2009; Reunanen et al. 2012). Also the plasmidic *spaCBA* from *L. paracasei* LOCK 0919 seems to be functional and responsible for the strong adhesiveness of this strain (Aleksandrzak-Piekarczyk et al. 2016). It is postulated that insertion of an IS element in the *spaC* promoter region in both *L. rhamnosus* GG and *L. paracasei* LOCK 0919 allows the expression of the pili genes (Douillard et al. 2013; Aleksandrzak-Piekarczyk et al. 2016). Notably, the upstream region of the IBB3423 *spaCBA* operon harbored by pLCAKO.2 also contains an insertion element indicating that it likely is functional. Indeed, the removal of pLCAKO.2 led to a marked decrease of IBB3423 hydrophobicity and adhesiveness to biotic and abiotic surfaces. The presence of such a plasmid should therefore facilitate colonization and ensure longer persistence in the host’s gut thereby conferring a competitive advantage over other bacteria. It can also increase the range of inhabited environments. Plasmidic localization of the *spaCBA* operon, such as in IBB3423, is extremely rare albeit not unique since among all the plasmids deposited at GenBank only two, pLOCK 0919 from *L. paracasei* LOCK 0919 (Koryszewska-Baginska et al. 2013; Aleksandrzak-Piekarczyk et al. 2016) and p1_LC355 from *L. paracasei* LC355 (NZ_CP029537.1), harbor the same pilus gene cluster.

In conclusion, the newly identified dairy strain *L. paracasei* subsp. *paracasei* IBB3423 presents genomic and functional features making it a very promising candidate probiotic. Owing to its high adhesiveness comparable to that of the benchmark strain *L. rhamnosus* GG and the ability to use a wide range of saccharides including inulin, it should be able to compete with other commercially applied strains. However, additional functional studies in vitro and in vivo should be performed to determine whether IBB3423 can persist in the gastrointestinal tract and exhibits health-promoting action. Last but not least, the isolation of this novel bacterial strain of such interesting characteristics is proof of the productiveness of citizen science.

## Electronic supplementary material

ESM 1 (PDF 629 kb)
