# Supplementary material for: Comparative genomics and functional analysis of a highly adhesive dairy Lactobacillus paracasei subsp. paracasei IBB3423 strain
Source: Appl Microbiol Biotechnol. 2019 Jul 29;103(18):7617–34. doi: 10.1007/s00253-019-10010-1 (PMC6717177; doi:10.1007/s00253-019-10010-1)
Supplement: Supplementary file 1 — (PDF 629 kb) [file 253_2019_10010_MOESM1_ESM.pdf]

Applied Microbiology and Biotechnology

Comparative genomics and functional analysis of a highly adhesive dairy *Lactobacillus paracasei* subsp. *paracasei* IBB3423 strain

Anna Koryszewska-Bagińska[1,2], Jan Gawor[1], Adriana Nowak[3], Marcin Grynberg[1,†], Tamara Aleksandrzak-Piekarczyk[1,*]

[1]Institute of Biochemistry and Biophysics, Polish Academy of Sciences (IBB PAS), Pawińskiego 5a, 02-106 Warsaw, Poland
[2]Present address: Department of Medical Biology, Medical University of Warsaw, Nowogrodzka 73, 02-118 Warsaw, Poland
[3]Institute of Fermentation Technology and Microbiology, Lodz University of Technology, Wolczanska 171/173, 90-924 Lodz, Poland

*Corresponding author. Mailing address: IBB PAS, Pawińskiego 5a, 02-106 Warsaw, Poland, Phone: (+48) 22 592 1213, Fax: (+48) 22 658 4636, E-mail: tamara@ibb.waw.pl

†Corresponding author for citizen science matters. Mailing address: IBB PAS, Pawińskiego 5a, 02-106 Warsaw, Poland, Phone: (+48) 22 592 2354, Fax: (+48) 22 658 4636, E-mail: greenb@ibb.waw.pl

Table S1. Number of IBB3423 genes assigned to general COG functional categories

COG functional class	№ ORFs	% ORFs*
**Metabolism**		
C - Energy production and conversion	100	3.13
E - Amino acid transport and metabolism	185	5.80
F - Nucleotide transport and metabolism	70	2.19
G - Carbohydrate transport and metabolism	273	8.56
H - Coenzyme transport and metabolism	46	1.44
I - Lipid transport and metabolism	55	1.72
P - Inorganic ion transport and metabolism	85	2.66
Q - Secondary metabolites biosynthesis, transport and catabolism	13	0.40
**Cellular processes and signaling**		
D - Cell division and chromosome partitioning	27	0.84
M - Cell envelope biogenesis, outer membrane	111	3.48
N - Cell motility	5	0.15
O - Posttranslational modification, protein turnover, chaperones	60	1.88
T - Signal transduction mechanisms	50	1.56
U - Intracellular trafficking, secretion, and vesicular transport	10	0.31
V - Defense mechanisms	65	2.03
**Information storage and processing**		
J - Translation, ribosomal structure and biogenesis	130	4.07
K - Transcription	191	5.98
L - DNA replication, recombination and repair	190	5.97
**Poorly characterized**		
R - General function prediction only	210	6.58
S - COG of unknown function	122	3.82
**Not in COGs**		
-	1190	37.31

*- based on the total number of ORFs in the IBB3423 genome

Table S2. Putative genes on plasmids pLCAKO.1 (A) and pLCAKO.2 (B). Pseudogenes are indicated by asterisk (*).

**A. pLCAKO.1 plasmid**						
**Locus_tag**	**gene**	**strand**	**CDS length (aa)**	**predicted product**	**aa overlap (identity) (%)**	**organism**
LCAKO_1p1	*repB*	+	352	replication initiation factor	100 (34)	*Staphylococcus pseudintermedius*
LCAKO_1p2	*orf2*	+	102	hypothetical protein		no hits
LCAKO_1p3	*orf3*	+	146	hypothetical protein	35 (28)	*Pseudomonas alkylphenolia*
LCAKO_1p4	*orf4*	+	50	hypothetical protein		no hits
LCAKO_1p5	*orf5*	+	642	hypothetical protein	43 (29)	*Enterococcus phoeniculicola*
LCAKO_1p6	*orf6*	+	83	hypothetical protein	33 (40)	*Enterococcus casseliflavus*
LCAKO_1p7	*orf7*	+	224	hypothetical protein.	124 (57)	*Streptococcus suis*
LCAKO_1p8	*orf8*	+	55	hypothetical protein		no hits
LCAKO_1p9	*orf9*	+	61	hypothetical protein		no hits
**B. pLCAKO.2 plasmid**						
**Locus_tag**	**gene**	**strand**	**CDS length (aa)**	**predicted product**	**aa overlap (identity) (%)**	**organism**
LCAKO_2p1	*repA*	+	352	replication protein	352 (100)	*Lactobacillus* spp.
LCAKO_2p2	*orf2*	+	192	hypothetical protein	192 (100)	*Lactobacillus* spp.
LCAKO_2p3	*orf3**	+		collagen adhesion protein		*Lactobacillus. paracasei*
LCAKO_2p4	*orf4*	-	96	transposase, IS5/IS1182 family	96 (100)	*Lactobacillus paracasei*
LCAKO_2p5	*orf5*	-	110	transposase	110 (100)	*Lactobacillus paracasei*
LCAKO_2p6	*orf6*	+	232	transposase	232 (100)	*Lactobacillus casei* group
LCAKO_2p7	*orf7*	+	235	hypothetical protein.	235 (100)	*Lactobacillus* spp.
LCAKO_2p8	*orf8*	-	66	hypothetical protein	66 (100)	*Lactobacillus* spp.
LCAKO_2p9	*orf9*	+	66	hypothetical protein	66 (100)	*Lactobacillus* spp.
LCAKO_2p10	*orf10*	+	87	hypothetical protein	86 (99)	*Lactobacillus* spp.
LCAKO_2p11	*or11*	+	50	hypothetical protein	38 (100)	*Lactobacillus* spp.
LCAKO_2p12	*orf12*	+	224	transposase, IS30 family	219 (100)	*Lactobacillus paracasei*
LCAKO_2p13	*orf13*	+	104	hypothetical protein	104 (100)	*Lactobacillus paracasei*
LCAKO_2p14	*orf14*	+	414	hypothetical protein	414 (100)	*Lactobacillus* spp.
LCAKO_2p15	*orf15*	+	83	hypothetical protein	83 (100)	*Lactobacillus paracasei*

LCAKO_2p16	*orf16*	+	845	hypothetical protein	843 (99)	*Lactobacillus* spp.
LCAKO_2p17	*orf17**	+		transposase		*Lactobacillus* spp.
LCAKO_2p18	*orf18*	-	338	transposase, IS30 family	338 (100)	*Lactobacillus* spp.
LCAKO_2p19	*orf19*	+	146	transposase, IS10 family	136 (93)	*Lactobacillus casei* group
LCAKO_2p20	*orf20*	-	57	hypothetical protein	57 (100)	*Lactobacillus casei* group
LCAKO_2p21	*orf21*	+	449	pyridine nucleotide-disulfide oxidoreductase	449 (100)	*Lactobacillus* spp.
LCAKO_2p22	*orf22*	+	53	hypothetical protein	53 (100)	*Lactobacillus* spp.
LCAKO_2p23	*orf23*	+	90	transposase	89 (100)	*Lactobacillus paracasei*
LCAKO_2p24	*orf24*	+	156	transposase, IS5/IS1182 family	155 (99)	*Lactobacillus.paracasei*
LCAKO_2p25	*orf25*	-	107	beta-1,3-glucosyltransferase	106 (100)	*Lactobacillus* spp.
LCAKO_2p26	*lacT*	+	292	beta-glucoside *bgl* operon antiterminator	292 (100)	*Lactobacillus* spp.
LCAKO_2p27	*lacE*	+	577	PTS system, lactose-specific IICB component	577 (100)	*Lactobacillus casei* group
LCAKO_2p28	*lacG*	+	474	6-phospho-beta-galactosidase	474 (100)	*Lactobacillus paracasei*
LCAKO_2p29	*lacF*	+	112	PTS system, lactose-specific IIA component	111 (100)	*Lactobacillus* spp.
LCAKO_2p30	*orf30*	+	42	hypothetical protein	33 (97)	*Lactobacillus coryniformis*
LCAKO_2p31	*orf31*	-	125	transposase	123 (98)	*Lactobacillus casei*
LCAKO_2p32	*orf32*	-	106	transposase, IS5 family	106 (100)	*Lactobacillus casei* group
LCAKO_2p33	*orf33*	-	185	serine acetyltransferase	185 (100)	*Lactobacillus* spp.
LCAKO_2p34	*orf34*	-	394	cystathionine gamma-lyase	394 (100)	*Lactobacillus* spp.
LCAKO_2p35	*orf35*	-	303	cystathionine beta-synthase	303 (100)	*Lactobacillus* spp.
LCAKO_2p36	*orf36*	+	41	hypothetical protein	18 (86)	*Lactobacillus plantarum*
LCAKO_2p37	*orf37*	+	231	hypothetical protein	221 (100)	*Lactobacillus* spp.
LCAKO_2p38	*orf38*	-	204	hypothetical protein	182 (89)	*Lactobacillus* spp.
LCAKO_2p39	*orf39*	-	63	hypothetical protein	63 (100)	*Lactobacillus paracasei*
LCAKO_2p40	*orf40*	+	232	transposase, IS30 family	231(99)	*Lactobacillus casei* group
LCAKO_2p41	*orf41*	-	66	hypothetical protein	65 (98)	*Lactobacillus* spp.
LCAKO_2p42	*orf42*	-	148	hypothetical protein	144 (97)	*Lactobacillus paracasei*
LCAKO_2p43	*orf43*	+	41	hypothetical protein	40 (98)	*Lactobacillus* spp.
LCAKO_2p44	*orf44*	+	40	hypothetical protein	39 (98)	*Lactobacillus paracasei*
LCAKO_2p45	*orf45*	+	399	transposase, IS110 family	399 (100)	*Lactobacillus* spp.
LCAKO_2p46	*or46*	+	39	hypothetical protein		no hits
LCAKO_2p47	*orf47*	+	297	DNA/RNA non-specific endonuclease	297 (100)	*Lactobacillus* spp.
LCAKO_2p48	*orf48*	+	119	hypothetical protein	118 (99)	*Lactobacillus* spp.

LCAKO_2p49	*orf49*	-	156	transposase, IS5/IS1182 family	152 (97)	*Lactobacillus rhamnosus*
LCAKO_2p50	*orf50*	-	106	transposase, IS5 family	105 (99)	*Lactobacillus casei* group
LCAKO_2p51	*spaC*	+	898	pilin minor subunit SpaC, LPXTG-anchored	877 (98)	*Lactobacillus* spp.
LCAKO_2p52	*spaB*	+	241	pilin minor subunit SpaB, LPXTG-anchored	240 (99)	*Lactobacillus casei*
LCAKO_2p53	*spaA*	+	334	pilin major protein SpaA, LPXTG-anchored	333 (99)	*Lactobacillus casei* group
LCAKO_2p54	*srtC*	+	359	class C sortase, LPXTG specific	359 (100)	*Lactobacillus rhamnosus*
LCAKO_2p55	*orf55*	+	52	hypothetical protein		no hits
LCAKO_2p56	*orf56*	-	132	pyridoxine 5'-phosphate oxidase V related flavin-nucleotide-binding protein	132 (100)	*Lactobacillus* spp.
LCAKO_2p57	*orf57*	-	123	hypothetical protein	123 (100)	*Lactobacillus* spp.
LCAKO_2p58	*orf58*	-	78	hypothetical protein	78 (100)	*Lactobacillus casei*
LCAKO_2p59	*orf59*	-	110	alcohol dehydrogenase	106 (96)	*Lactobacillus paracasei*
LCAKO_2p60	*orf60*	-	94	hypothetical protein	93 (99)	*Lactobacillus* spp.
LCAKO_2p61	*orf61*	-	216	pin-related site recombinase/DNA invertase	215 (99)	*Lactobacillus* spp.
LCAKO_2p62	*orf62*	+	229	transposase, IS30 family	229 (100)	*Lactobacillus paracasei*
LCAKO_2p63	*orf63*	-	604	beta-N-acetylglucosaminidase	604 (100)	*Lactobacillus* spp.
LCAKO_2p64	*orf64**	+		DNA polymerase		*Lactobacillus paracasei*
LCAKO_2p65	*orf65*	+	77	hypothetical protein	67 (93)	*Lactobacillus paracasei*
LCAKO_2p66	*orf66*	-	132	hypothetical protein.	132 (100)	*Lactobacillus* spp.
LCAKO_2p67	*parA*	-	269	chromosome/plasmid partitioning ATPase	269 (100)	*Lactobacillus* spp.

Table S3. Carbohydrate transporters encoded in IBB3423 genome. (A) PTS permeases, (B) ABC transporters, (C) MFS sugar secondary transporters.

A. PTS permeases

Locus tags	Specificity
LCAKO_0120<br>LCAKO_1279<br>LCAKO_2383<br>LCAKO_3203	beta -glucoside
LCAKO_0277<br>LCAKO_0305<br>LCAKO_0525<br>LCAKO_1982<br>LCAKO_2269<br>LCAKO_3108	cellobiose
LCAKO_2p27	lactose
LCAKO_0688	trehalose
LCAKO_2297	sucrose
LCAKO_0369<br>LCAKO_3004<br>LCAKO_3023<br>LCAKO_3122	mannose
LCAKO_3056	mannose/fructose/*N*-acetylgalactosamine
LCAKO_0299	galactosamine
LCAKO_0448	fructose/mannose
LCAKO_0399<br>LCAKO_0404<br>LCAKO_1552<br>LCAKO_2892	fructose
LCAKO_0409	fructose/mannitol
LCAKO_0458<br>LCAKO_3189	mannitol
LCAKO_0738	galactose
LCAKO_2902<br>LCAKO_2918<br>LCAKO_2955	galacitol
LCAKO_0351<br>LCAKO_2967<br>LCAKO_2996	ascorbate
LCAKO_0426<br>LCAKO_0479	sorbose
LCAKO_0386<br>LCAKO_0413	gluconate
LCAKO_2986	glucitol/sorbitol

B. ABC transporters

Locus tag	Specificity
LCAKO_1150<br>LCAKO_1152<br>LCAKO_1153<br>LCAKO_1155	maltose/maltodextrin
LCAKO_0327<br>LCAKO_0328<br>LCAKO_0329<br>LCAKO_0330<br>LCAKO_2269<br>LCAKO_3108	ribose
LCAKO_3042<br>LCAKO_3043<br>LCAKO_3044<br>LCAKO_3045	glycerol
LCAKO_0290<br>LCAKO_1161<br>LCAKO_1162<br>LCAKO_1163<br>LCAKO_1164	multiple sugars

C. MFS sugar secondary transporters

Locus tag	Specificity
LCAKO_1302	glucose
LCAKO_2018	melibiose
LCAKO_0230<br>LCAKO_1963	polyol
LCAKO_0438<br>LCAKO_0488<br>LCAKO_0506<br>LCAKO_1002<br>LCAKO_1137<br>LCAKO_1626<br>LCAKO_2475<br>LCAKO_2543<br>LCAKO_2601<br>LCAKO_3016	uncharacterized

Table S4. Potential genetic determinants of *L. paracasei* subsp. *paracasei* IBB3423 adhesive capacities (Cell wall-anchored or surface-associated adhesins).

	Predicted product	Length [aa]	SPI	Extracellular matrix (ECM) proteins-binding domains	Accession no.	Cell surface linkage	Accession	Subcellular localization prediction
**CHROMOSOME**								
LCAKO_0030	Putative peptidoglycan binding domain protein	716	NO			PG_binding_1	pfam01471	Unknown
LCAKO_0047	Endo-N-acetylmuramidase	716	NO			PG_binding_1	pfam01471	Unknown
LCAKO_0110	Leucine-rich repeat C-terminal cell-wall surface anchor repeat MucBP damain protein	3254	NO	MucBP<br>LRR_4	pfam06458<br>pfam12799	C-term_anchor	pfam13461	Cell wall/Extracellular
LCAKO_0113	LPXTG-motif cell wall anchor domain protein	507	NO			LPXTG	TIGR01167	Cell wall
LCAKO_0160	WxL domain cell surface protein	684	SPI			WxL	pfam13731	Unknown
LCAKO_0162	WxL domain cell surface protein	257	SPI			WxL	pfam13731	Non-Cytoplasmic
LCAKO_0163	WxL domain cell surface protein	248	SPI			WxL	pfam13731	Non-Cytoplasmic
LCAKO_0451	KxYKxGKxW signal peptide and MucBP domain beta-fructosidase	1296	KxYKxGKxW	MucBP<br>Big_3 5x	pfam06458<br>pfam07523	LPXTG;<br>C-term_anchor	TIGR01167;<br>pfam13461	Cell wall
LCAKO_0469	N-acetylmuramoyl-L-alanine amidase	254	NO			LysM	cd00118	Unknown
LCAKO_0529	Pilin minor subunit SpaC	909	SPI	Cna_B 2x<br>CollagenBindB<br>vWFA	pfam05738<br>cd00222<br>cd00198	LPXTG	TIGR01167	Cell wall
LCAKO_0530	Pilin minor subunit SpaB	268	SPI	GramPos_pilinD1	pfam16555	LPXTG	TIGR01167	Cell wall
LCAKO_0531	Pilin major protein SpaA	317	NO	GramPos_pilinD1	pfam16555	LPXTG	TIGR01167	Cell wall
LCAKO_0532	Class C sortase, LPXTG specific	358	NO					Membrane
LCAKO_0541	KxYKxGKxW signal peptide cell-wall-anchored proteinase	2231	KxYKxGKxW			LPXTG	TIGR01167	Cell wall
LCAKO_0596	Chitinase	519	SPI	Big_3 2x	pfam07523			Non-Cytoplasmic
LCAKO_0636	KxYKxGKxW signal peptide, MucBP and legume-lectin domains-containing cell surface protein, LPXTG-anchored	874	KxYKxGKxW	MucBP<br>lectin_L-type	pfam06458<br>cd01951	LPXTG;<br>C-term_anchor	TIGR01167;<br>pfam13461	Cell wall
LCAKO_0658	Phage lysin, 1,4-beta-*N*-acetylmuramidase	323	NO			LysM	cd00118	Unknown
LCAKO_0665	WxL domain-containing cell surface protein	222	SPI			WxL	pfam13731	Non-Cytoplasmic
LCAKO_0668	Legume-lectin and WxL domains-containing surface protein	665	SPI	lectin_L-type	cd01951	WxL	pfam13731	Non-Cytoplasmic
LCAKO_0670	WxL domain-containing cell surface protein	233	SPI			WxL	pfam13731	Non-Cytoplasmic
LCAKO_0672	WxL domain-containing cell surface protein	234	SPI			WxL	pfam13731	Membrane
LCAKO_0673	Legume-lectin and WxL domains-containing surface protein	674	SPI	lectin_L-type	cd01951	WxL	pfam13731	Non-Cytoplasmic
LCAKO_0985	Bacterial group 2 Ig-like protein	71	NO	BID_2	smart00635			Extracellular
LCAKO_1081	DNA translocase FtsK	773	NO					Membrane
LCAKO_1428	LysM peptidoglycan-binding protein	522	SPI			LysM 3x	cd00118	Extracellular

	Predicted product	Length [aa]	SPI	Extracellular matrix (ECM) proteins-binding domains	Accession no.	Cell surface linkage	Accession	Subcellular localization prediction
LCAKO_1580	LysM peptidoglycan-binding protein	206	NO			LysM	cd00118	Unknown
LCAKO_1598	Carboxyl-terminal protease	461	SPI			PG_binding_1	pfam01471	Membrane
LCAKO_1644	Fibronectin/fibrinogen-binding protein	567	NO	FbpA	pfam05833			Cytoplasmic
LCAKO_2029	Hypothetical protein containing MucBP domain	170	NO	MucBP	pfam06458			Unknown
LCAKO_2107	LysM domain surface protein	1140	SPI			LysM 4x	cd00118	Cell wall/Extracellular
LCAKO_2111	Surface protein	531	SPI			LPXTG	TIGR01167	Cell wall
LCAKO_2118	Cna adhesin type-B domain, LPXTG-anchored	1269	SPI	Cna_B 5x	pfam05738	LPXTG	TIGR01167	Cell wall
LCAKO_2183	Lysozyme M1, 1,4-beta-*N*-acetylmuramidase	693	SPI			SH3_8 4x	pfam13457	Extracellular
LCAKO_2218	Flagellar hook-length control protein FliK	300	SPI					Non-Cytoplasmic
LCAKO_2275	WxL domain secreted protein	201	SPI			WxL	pfam13731	Non-Cytoplasmic
LCAKO_2334	Class A sortase, LPXTG specific	233	NO					Membrane
LCAKO_2519	KxYKxGKxW signal peptide and MucBP domain surface protein	423	KxYKxGKxW	MucBP 3x	pfam06458	LPXTG; C-term_anchor 3x	TIGR01167; pfam13461	Cell wall
LCAKO_2549	Class C sortase, LPXTG specific	275	SPI					Membrane
LCAKO_2550	Pilus major protein SpaD	519	SPI	Cna_B<br>GramPos_pilinD1<br>RrgB_K2N_iso_D2	pfam05738<br>pfam16555<br>TIGR04226	LPXTG	TIGR01167	Non-Cytoplasmic
LCAKO_2551	Pilus specific minor protein SpaE	440	SPI			LPXTG	TIGR01167	Cell wall
LCAKO_2552	Pilus minor protein SpaF	1001	SPI	Cna_B 2x	pfam05738	LPXTG	TIGR01167	Cell wall
LCAKO_2606	Manganese ABC transporter, substrate-binding protein SitA	313	SPI	ZnuA	pfam01297			Membrane
LCAKO_2612	Zinc ABC transporter, substrate-binding protein ZnuA	305	SPII	ZnuA	pfam01297			Membrane
LCAKO_2618	Cna type-B domain collagen binding protein, LPXTG-anchored	598	SPI	Cna_B<br>Collagen_bind	pfam05738<br>pfam05737	LPXTG	TIGR01167	Cell wall
LCAKO_2619	Zinc ABC transporter, substrate-binding protein ZnuA	300	SPII	ZnuA	pfam01297			Membrane
LCAKO_2625	Flagellar hook-length control protein FliK	845	SPI					Non-Cytoplasmic
LCAKO_2652	Cna type-B domain adhesin	1269	SPI	Cna_B 6x	pfam05738	LPXTG	TIGR01167	Cell wall
LCAKO_2745	Cna type-B domain adhesin	2197	NO	Cna_B 16x	pfam05738	LPXTG	TIGR01167	Cell wall
LCAKO_2769	WxL domain cell surface complex protein precursor	299	SPI			WxL	pfam13731	Non-Cytoplasmic
LCAKO_2773	Legume-lectin, WxL and MucBP domains-containing cell surface protein	821	SPI	MucBP<br>lectin_L-type	pfam06458<br>cd01951	WxL	pfam13731	Membrane
LCAKO_2784	L,D-transpeptidase / peptidoglycan binding protein	456	SPI			PG_binding_4 2x	pfam12229	Unknown

	Predicted product	Length [aa]	SPI	Extracellular matrix (ECM) proteins-binding domains	Accession no.	Cell surface linkage	Accession	Subcellular localization prediction
LCAKO_2859	Phosphinothricin *N*-acetyltransferase	166	NO			LysM	cd00118	Unknown
LCAKO_2941	Polysaccharide lyase	1029	SPI			LPXTG	TIGR01167	Cell wall
LCAKO_3201	KxYKxGKxW signal peptide leucine-rich repeat adhesion exoprotein,	1983	KxYKxGKxW	LRR_5 3x	pfam13306	LPXTG	TIGR01167	Cell wall
**PLASMID 2**								
pLCAKO.2_51	Pilin minor subunit SpaC	898	SPI	CollagenBindB<br>vWFA<br>Cna_B 2x	cd00222<br>cd00198<br>pfam05738	LPXTG	TIGR01167	Cell wall
pLCAKO.2_52	Pilin minor subunit SpaB	241	SPI	GramPos_pilinD1	pfam16555	LPXTG	TIGR01167	Cell wall
pLCAKO.2_53	Pilin major protein SpaA	334	SPI	GramPos_pilinD1	pfam16555	LPXTG	TIGR01167	Cell wall
pLCAKO.2_54	Class C sortase, LPXTG specific	359	NO					Unknown
pLCAKO.2_63	Beta-*N*-acetylglucosaminidase	641	SPI			LPXTG	TIGR01167	Cell wall

pfam06458 (MucBP): mucine-binding protein domain
pfam05738 (CnaB): Cna protein B-type domain
cd00222 (collagenBindB): repeat unit of collagen-binding protein domain B
pfam05737 (collagen_bind): collagen binding domain
pfam05833 (FbpA): fibronectin-binding protein A N-terminus
pfam12799 (LRR_4): leucine rich repeats (2 copies)
pfam13306 (LRR_5): leucine rich repeats (6 copies)
smart00635 (BID_2): bacterial Ig-like domain (group 2)
pfam07523 (Big_3): bacterial Ig-like domain (group 3)
cd01951 (lectin_L-type): legume lectins
cd00198 (vWFA): von Willebrand factor type A (vWA) domain
pfam01297 (ZnuA): zinc-uptake complex component A periplasmic
pfam16555 (GramPos_pilinD1): Gram-positive pilin subunit D1, N-terminal
TIGR04226 (RrgB_K2N_iso_D2): fimbrial isopeptide formation D2 domain
pfam13731 (WxL): WxL domain surface cell wall-binding
pfam01471 (PG_binding_1): putative peptidoglycan binding domain
pfam13461 (C-term_anchor): cell-wall surface anchor repeat
pfam12229 (PG_binding_4): putative peptidoglycan binding domain
TIGR03715 (KxYKxGKxW): KxYKxGKxW signal peptide
TIGR01167 (LPXTG_anchor): LPXTG-motif cell wall anchor domain
cd00118 (LysM): peptidoglycan binding Lysine Motif domain

Table S5. General genome features of completely sequenced *L. casei* and *L. paracasei* strains.

Strain	Size (Mbp)	GC%	CDS	Origin
Lactobacillus casei				
L. casei BL23	3.079	46.30	3029	Cheese
L. casei 12A	2.907	46.40	2801	Corn silage
L. casei W56	3.132	46.25	3010	Probiotic products
L. casei subsp. *casei* ATCC 393	2.952	47.86	2636	Cheese
L. casei LC5	3.132	47.90	2893	Commercial dietary supplements
Lactobacillus paracasei				
L. paracasei subsp. *paracasei* IBB3423	3.240	46.30	3189	Bovine milk
L. paracasei Zhang	2.898	46.46	2762	Koumiss
L. paracasei BD-II	3.127	46.25	3298	Koumiss
L. paracasei LOCK 0919	3.143	46.18	3079	Healthy child's feaces
L. paracasei LC2W	3.077	46.35	3002	Dairy product
L. paracasei HDS-01	3.038	46.40	2855	Chinese sauerkraut
L. paracasei ATCC 334	2.924	46.56	2764	Swiss cheese
L. paracasei subsp. *paracasei* JCM 8130	3.078	46.56	2921	Dairy product
L. paracasei subsp. *paracasei* 8700:2	3.025	46.30	2917	Human colonic mucosa
L. paracasei N1115	3.064	46.46	2952	Dairy product
L. paracasei CAUH35	2.973	46.33	2777	Koumiss
L. paracasei L9	3.076	46.30	2926	Human centenarian's feaces
L. paracasei KL1	2.918	46.60	2793	Milk
L. paracasei IIA	3.246	46.22	3116	Beef
L. paracasei TK1501	2.942	46.50	2776	Congee
L. paracasei FAM18149	2.969	46.34	2828	Cheese
L. paracasei subsp. *paracasei* TMW 1.1434	3.170	46.32	2898	Human small intestine
L. paracasei HD1.7	3.173	46.40	2819	Chinese sauerkraut
L. paracasei EG9	3.074	46.44	2945	Cheese
L. paracasei Lpc10	3.052	46.30	2933	Merlot wine
L. paracasei LC355	3.143	46.41	3247	Cream products

Figure S1. **Adherence of *Lactobacillus* strains to microtiter PS plates.** Data represent means from five to nine repeats (±SD).

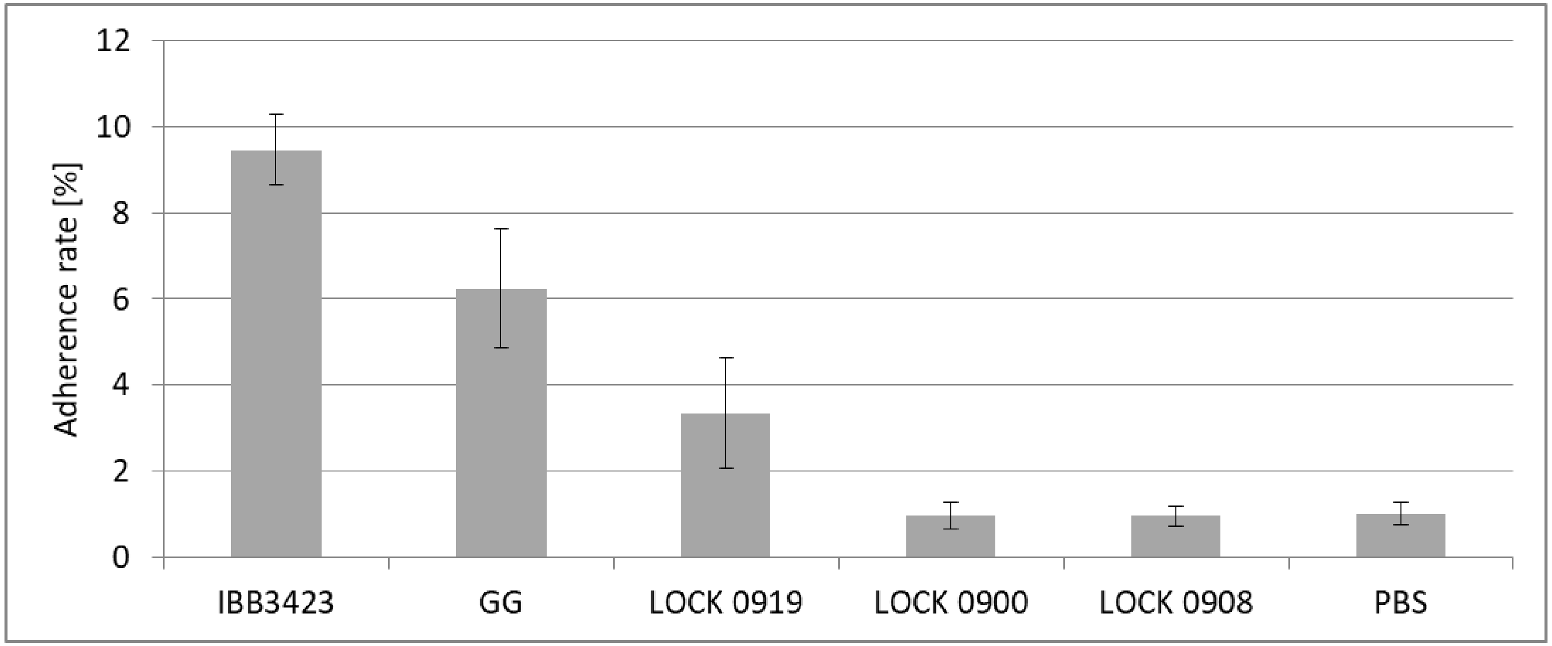

Information S6. **The following persons were involved in the collection of milk samples and thier initial analysis within the citizen science project:** Araśniewicz Julita, Argalska Klaudia, Bagińska Weronika, Bakuła Klaudia, Bałdyga Artur, Baniewska Dorota, Barczak Piotr, Bauman Katarzyna, Bednarczyk Kinga, Bednarczyk Piotr, Białkowska Dominika, Biedruń Izabela, Bińczak Agata, Błachowicz Aleksandra, Błaziak Patrycja, Błażewicz Grzegorz, Bogdanowicz Julia, Bohdanowicz Denis, Borowski Marek, Borys Patrycja, Bronakowska Paulina, Brykała Anna, Bucholski Tomasz, Cabaj Anna, Chamyga Dominika, Chamyga Weronika, Chojnowska Magdalena, Choromuńska Weronika, Chudzik Julia, Cisz Wiktoria, Czech Maja, Czechowska Michalina, Czławski Robert, Czuba Bartosz, Daczyńska Maja, Darmofał Dawid, Dąbkowska Anna, Dej Edyta, Demarko Paulina, Dembna Dominika, Deptuła Sebastian, Domian Michał, Doroszkiewicz Małgorzata, Dreksler Ola, Drozd Dorota, Duda Alicja, Dudko Anna, Dydykało Oliwia, Falkowska Dagmara, Fiuk Sebastian, Folga Dominika, Folga Natalia, Gałęska Kamila, Gedig Jolanta, Góralska Marta, Grabowska Milena, Gromadzki Dominik, Grzelak Małgorzata, Grzywacz Urszula, Gulewicz Karolina, Gwozdowicz Katarzyna, Hetman Joanna, Jackowski Wojciech, Jakubisiak Monika, Jankowska Klaudia, Jasiulewicz Aleksandra, Jóźwika Paulina, Kadzewicz Klaudia, Kalwara Martyna, Kamińska Agnieszka, Kaniuk Szymon, Kaszuba Agnieszk, Kawecka Ewa, Kawecka Iwona, Kempisty Justyna, Kij Zuzanna, Kolos Magdalena, Koprucki Piotr, Korowaj Ewelina, Kowalczyk Aleksandra, Kowalczyk Dawid, Kowalczyk Ernest, Kowalczyk Karol, Kowalska Marlena, Kozłowska Marta, Krasilewicz Oliwia, Kreft Natalia, Król Elżbieta, Królicki Dominik, Królikiewicz Igor, Kunka Natalia, Kurek Marta, Kuryło Kasia, Kurzawska Hanna, Kuśmierczyk Kasandra, Lasowska Alicja, Ledzińska Adrianna, Lewandowska Bożena, Lewandowska Martyna, Ludkiewicz Piotr, Łęczkowski Sebastian, Łobaz Kamila, Łokucjewska Anna, Łowczyk Wojciech, Łukaszuk Klaudia, Maciejewska Beata, Maciejewska Monika, Malczyk Ewa, Mańka Wiktoria, Matusiak-Ordon Małgorzata, Mazuchowski Michał, Michalska Małgorzata, Michałowska Agnieszka, Michałowska Karolina, Michna Aleksandra, Mikulska Paulina, Milewicz Magda, Młynarski Mateusz, Moskiewicz Natalia, Moszczyńska Aleksandra, Murach Klaudia, Myszkowska Paulina, Najmuła Kamil, Narkiewicz Zuzanna, Naumaniez Adam, Nawrocka Alicja, Nieściór Julia, Nowacka Zuzanna, Nowak Patrycja, Obrębska Patrycja, Ostoja-Lniski Robert, Pałuska Karolina, Paszkowska Aleksandra, Piotrak Karolina, Piotrowska Anna Piskorz Wiktoria, Podpora Michał, Popis Gabriela, Poterała Paulina, Potrapeluk Katarzyna, Powierża Laura, Poźmirowska Weronika, Puczel Mateusz, Radziszewska Alina, Roguszka Karolina, Rokicka Adrianna, Romanowski Michał, Rosłon Adrianna, Rudzińska Urszula, Rudzka Katarzyna, Rzosińska Andżelika, Sitarski Jakub, Siwkowska Natalia, Skoczeń Joanna, Smolińska Natalia, Sobieraj Agnieszka, Sobieraj Monika, Sobolewska Agnieszka, Sobolewska Julia, Sobotka Monika, Sokulska Maria, Soliwoda Aleksandra, Stankiewicz Kuba, Staszak Krzysztof, Stemplewska Natalia, Stepaniuk Monika, Stepnowska Zuzanna, Stopyra Justyna, Suchocka Zuzanna, Suchodolska Anna, Szczepkowska Elżbieta, Szczeszak Agnieszka, Szlachcic Karolina, Szut Jakub, Szydlik Aleksandra, Szymczak Dominika, Szyszkowska Klaudia, Tałan Mateusz, Tarasiewicz Justyna, Tomaszewska Joanna, Trzeciak Jakub, Tucholska Joanna, Tur Beata, Tyc Karol, Ustianowska Julia, Walut Agnieszka, Wasilewska Ola, Waśniewska Aleksandra, Wdowska Martyna, Węgrzyn Damian, Wielga Aleksandra, Wielgosz Edyta, Włodarska Eliza, Włodarska Kamila, Wojciechowska Patrycja, Wojtkowiak Kacper, Wolska Dominika, Wołodźko Mateusz, Wójtowicz Aniela, Wasilewska Joanna, Wyszyńska Hanna, Zaboroś Joanna, Zarzycki Jacek, Zawalich Paulina, Zdziarska Adrianna, Zieliński Arkadiusz, Zwoliński Beniamin, Zyskowski Adrian.
